# Assessment of Energy Effects Determining *cis*-*trans* Proline Isomerization in Dipeptides

**DOI:** 10.1021/acsphyschemau.5c00072

**Published:** 2025-12-04

**Authors:** Natalia Díaz, Roberto López, Ángel Martín-Pendás, Dimas Suárez

**Affiliations:** † Departamento de Química Física y Analítica, Facultad de Química, 16763 Universidad de Oviedo, Avda. Julián Clavería 8, 33006 Oviedo, Asturias, Spain; ‡ Departamento de Química y Física Aplicadas, Facultad de Biología, 16762 Universidad de León, Campus de Vegazana s/n, 24071 León, Castilla y León, Spain

**Keywords:** peptide bond, *cis*−*trans* isomerization, quantum chemical calculations, energy decomposition analysis, free energy, molecular dynamics

## Abstract

Herein, we present the results of molecular dynamics, potential of mean force (PMF) and quantum mechanical (QM) calculations aimed to investigate the *cis–trans* equilibria of short peptides: capped Ac-Z-NHMe, Ac-X-Z-NHMe, and *zwitterionic* Leu-Z with X = Gln, Leu, Tyr and Z = Pro, Ala. Both PMF free energies and average QM energies in aqueous solution consistently predict that the Ala *→* Pro substitution stabilizes the Ac/X-Z *cis* isomer in all the model compounds. Using the interacting quantum atoms method, we decomposed the average QM energies into physical components and performed a comparative analysis between the Pro-containing peptides and their Ala-substituted counterparts. The results point out that *cis–trans* isomerization is not controlled by a single steric or electronic contribution and unveil a mixture of electrostatic, steric and hyperconjugative effects that is modulated by the dipeptide sequence. It is also shown that solute–solvent interactions stabilize systematically the *trans* and *cis* isomer of the Ala- and Pro-containing capped peptides, respectively, suggesting thus that solvation plays a key role in the Pro *cis* effect observed in these systems in agreement with former proposals.

## Graphical abstract

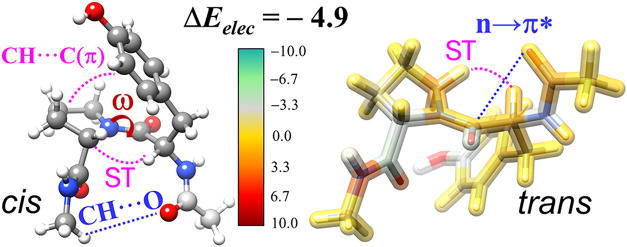

## Introduction

Intrinsically Disordered Proteins (IDPs) can adopt various secondary and tertiary structures, yet they play a fundamental role in biological processes such as cell cycle regulation, DNA damage repair, T-cell activation, ion channel gating, predisposition to certain diseases, or mutation prevalence.^1,2^ The relevance of the 20 natural amino acids for IDP behavior has been studied experimentally and computationally for years. As a result, amino acids are usually classified into “order-promoting” (Cys, Trp, Tyr, Ile, Phe, Val, Leu, His, Thr, and Asn) and “disorder promoting”—or IDP-favoring—residues (such as Asp, Met, Lys, Arg, Ser, Gln, Glu and Pro).^3^ Among the latter, proline has garnered much special attention since the early studies of Richard Willstätter in 1900 ^4^ due to its greatest potential to favor IDPs.

Proline is unique among natural amino acids due to the presence of a five membered pyrrolidine ring formed by the N–Cα backbone moiety and the Cδ–Cγ–Cβ side chain.^1,5^ This cyclic structure drastically restricts rotation around the N–Cα bond (φ angle) although the puckering of the pyrrolidine ring introduces some flexibility. Furthermore, X-Pro peptide bonds lack the amide proton at neutral pH, so hydrogen bonding interactions are restricted to the carbonyl group. As a result, prolines have a significant impact on the conformation of peptides and proteins, behaving as disruptors of secondary elements and favoring structures such as the polyproline type II (PPII) helix characteristic of multiproline sequences.^6^ These proline-rich regions are particularly relevant in the proteome of certain viruses.^7^

Prolines are considered as functionally relevant amino acids. The pyrrolidine ring in proline makes the *cis*–*trans* equilibrium of X-Pro bonds especially distinct from that observed in other peptide bonds.^8^ For instance, the analysis of a nonredundant data set of 571 protein crystal structures showed that only 0.03% of X-nonPro peptide bonds adopt the *cis* isomer, whereas this prevalence increases to 5% in X-Pro bonds.^9,10^ Other studies have reported higher populations of *cis*-Pro in short peptides such as the Ac-Ala-X-Pro-Ala-Lys-NH_2_ sequence for which nuclear magnetic resonance (NMR) experiments provided X-Pro *cis* contents ranging from 6.0 to 37.3% depending on the identity of the X amino acid.^11^ In general, *cis*–*trans* X-Pro isomerization affects protein folding, stability, and function^2,12^ and, accordingly, various human diseases have been linked to the dysregulation of X-Pro isomerization.

The number of possible isomers in peptide molecules with *n* prolines rises to 2^*n*^ so that the study of *cis*–*trans* isomerization in large proteins with X-Pro or X-Pro-Pro linkages is still a challenge.^12^ Consequently, focus has been placed on computational calculations and experimental studies using X-ray diffraction or NMR spectra for the analysis of small oligopeptides.^12^ These studies have shown that proline has a similar energy for the *cis* and *trans* X-Pro isomers.^1,13,14^

Traditionally, steric effects associated with the −CαH– positions of consecutive amino acids that can be in clashing proximity in the *cis* isomer of peptide bonds, have been considered as responsible for the greater prevalence of the *trans* peptide bond in proteins.^1,2,15^ The Pro *cis* effect, that is, the stabilization of the *cis* isomer in X-Pro linkages, would be the consequence of similar steric hindrance in both the *cis-*and *trans* isomers. However, the analysis of small systems has elucidated other factors that can regulate *cis*–*trans* isomerization. For example, Hinderaker and Raines^5^ have provided experimental evidence that steric effects alone cannot account for the *trans* preference of peptide bonds in *N*-formyl-l-proline systems. Thus, electronic effects arising from hyperconjugative delocalization of a nonbonding electron pair (*n*) from a carbonyl oxygen to the π* orbital of an adjacent C=O have been proposed to stabilize the *trans* isomer by 0.7 kcal/mol.^5^ This *anomeric* effect is only possible in *trans* conformations and may be especially significant in prolyl residues due to the pyrrolidine structure. Similarly, experimental and computational studies by Newberry et al. on proline model systems have quantified the energy of the *n* → π* interaction by at least 0.27 kcal/mol.^16^

The equilibrium *cis*–*trans* ratio (*K*_*trans*/*cis*_) in the proline containing peptides can also be influenced by the nature of the residues bonded to proline as computed by Kang et al. on a series of Ac-X-Pro-NHMe dipeptides (X = all 20 natural amino acids),^14^ reporting a varying (0.7–6.1%) population of *cis* isomers. Han and Kang conducted a similar computational analysis in Ac-Pro-X-NHMe dipeptides (X = Ala, Leu, Val, Gly, Cys, Met, Phe, Tyr, Asn, Asp, and Ser), quantifying the importance of the medium polarity.^17^ This observation has been refined by Siebler et al. in Ac-Pro-OMe and Ac-Pro-NHMe_2_ systems,^18^ who claim that solvent polarity effects are in fine balance with *n* → π* interactions. The effects of temperature, solvent, and pH have been also studied by Lee et al. in Ac-Pro-Gly-Pro.^19^ The identity of the preceding and following residues around an X-Pro linkage can be responsible for the presence of 4–30% *cis* isomers in IDPs, as suggested by Sebák et al.,^12,13^ who relied on the Grathwohl and Wüthrich’s 1976 study on the effect of polypeptide chain configuration in X-Pro systems (X = Gly, Ala, Leu, Phe)^20^ through the protonation state of dipeptides and the identity of the solvent. According to these results, aromatic residues favor the *cis* isomer in X-Pro peptide bonds. This effect would result from a favorable CH/π interaction between proline and the aromatic ring in the *cis* isomer.^21,22^

As noticed in the literature, steric, electronic and solvent effects (among others) may all be behind the isomeric preferences observed in X-Pro peptide bonds. However, direct comparisons among these factors are largely hampered by the diversity of experimental and/or computational methodologies that have been employed in the former studies. Therefore, we aim in this work to reexamine the *cis* effect exerted by Pro in dipeptides using a common computational methodology, what may be a prerequisite for understanding *cis*–*trans* isomerization in more complex biomolecules. Thus, we present a comparative analysis of the thermodynamic *cis*–*trans* preference in aqueous solution for the following Ac/NHMe-capped peptides, Ac-Z-NHMe, Ac-Gln-Z-NHMe, Ac-Leu-Z-NHMe, and Ac-Tyr-Z-NHMe, and one *zwitterionic* Leu-Z dipeptide where Z can be Ala or Pro. For each model system, we performed first molecular dynamics (MD) and potential of mean force (PMF) calculations in explicit solvent that characterize the structure and energetic stability of the *cis*-*trans* isomers. Subsequently, to better appreciate the relative weight of the small energy effects influencing the *cis*–*trans* equilibrium, we carried out quantum mechanical (QM) calculations including solvent effects on hundreds of conformers as extracted from the equilibrium MD trajectories. The average values of the QM *cis*–*trans* energies were subject to a detailed decomposition analysis using the Interacting Quantum Atoms (IQA)^23^ method that has been developed in the context of the quantum theory of atoms in molecules (QTAIM). IQA relies on the real space partitioning into the attraction (atomic) basins (Ω_I_) of the gradient field of the QM electron density and provides thereby atomic energies *E*(Ω_I_) and diatomic energies *E*(Ω_I_,Ω_J_). Compared with other energy decomposition analysis (EDA) methods, the IQA approach has the advantage of dissecting either intermolecular or intramolecular energy differences into various QM and classical electrostatic contributions.^24,25^ Furthermore, IQA can easily absorb continuum-solvent effects into atomic net energies,^26^ allowing thus to assess the importance of solute–solvent interactions. In this way, the IQA methodology will allow us to treat different energetic terms (steric, electronic, ···) on the same basis, yielding thus a full energetic description of molecular properties influencing the *cis*–*trans* isomeric preference of Pro-containing dipeptides.

## Methods

### Molecular Dynamics and Free Energy Calculations

Initial coordinates for the dipeptide molecules were built using the tLEaP program included in the Amber22 package.^27,28^ The initial structures were surrounded by an octahedral box of water molecules that extended at least 16 Å from the solute atoms. The molecular mechanics (MM) representation consisted of the physics-based Amber ff19SB force field^29^ for the peptide atoms and the OPC four-point water model.^30^ The *cis* and *trans* configuration of the X-Pro and X-Ala peptide bonds were considered.

Energy minimization and MD simulations were carried out using the SANDER and PMEMD programs included in Amber22. The water molecules were initially relaxed by means of energy minimizations and 200 ps of MD. Then the full systems were minimized and heated gradually to 300 K carrying out 60 ps of constant volume (NVT) MD with a 1 fs time step. Subsequently, the density was adjusted by means of 2.0 ns of constant pressure (NPT) MD with a 2 fs time step and using the Monte Carlo barostat as implemented in PMEMD. Langevin dynamics was employed to control the temperature (300 K) with a collision frequency of 2 ps^–1^. The SHAKE algorithm was employed to constrain all R–H bonds, and periodic boundary conditions were applied to simulate a continuous system. A nonbonded cutoff of 9.0 Å was used whereas the Particle-Mesh-Ewald method was employed to include the contributions of long-range interactions. The GPU accelerated version of the PMEMD code^31^ was employed in the MD production runs. The production phase of the MD simulations was extended up to 500 ns and coordinates were saved for analysts every 2.5 ps. The coordinates of the solute atoms along the MD trajectories were clustered using the CPPTRAJ module^32^ of Amber22. Other structural descriptors such as the puckering angle of the pyrrolidine ring, and the radius of gyration were also calculated with CPPTRAJ.

### Umbrella Sampling and Potential of Mean Force Calculations

The dihedral angle ω of the amide linkage of interest (see Scheme 1) was taken as the reaction coordinate and the periodic interval [0, 360°] was divided into subintervals (windows) defined by 120 intermediate values ω_0,*i*_ located at identical spacings of δ=3 degrees (0.052 rad). A harmonic potential centered at each ω_0,*i*_ value (*i.e*., *E*_*i*_ = *K*(ω – ω_0,*i*_)^2^) was employed to bias the sampling within the associated window. We assigned a value to the force constant of *K* = 300 kcal mol^–1^ degree^–2^, which ensured a smooth overlap of the distribution of the ω values between neighboring windows.

**Scheme 1:**
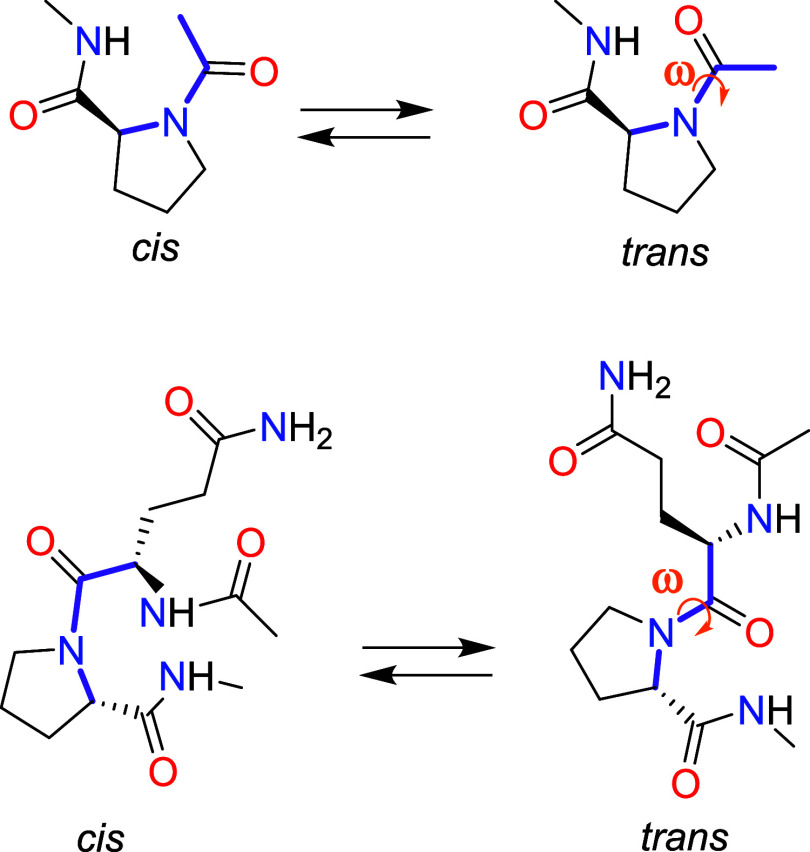
*cis-trans* Isomerization Equilibria

The umbrella sampling (US) simulations were performed using the ABMD code^33^ implemented in the SANDER program. Initial coordinates were taken from the last snapshot of the previous MD trajectories while the end point of each US window was used as a starting point for the next. Each window consisted of 1 ns of equilibration followed by 4 ns of production dynamics. The value of the reaction coordinate ω was saved every 2.5 ps.

The potential of mean force (PMF) was derived using the Histogram Analysis method (WHAM),^34^ imposing the periodicity of the reaction coordinate and selecting a small value for the convergence tolerance (0.00001 kcal/mol). The statistical uncertainty of the free energy profile was assessed using the Montecarlo bootstrap method implemented in WHAM, resulting in small errors (<0.1 kcal/mol). The free energy difference was obtained from the accumulated Boltzmann probabilities of observing the *trans* (*P*_*trans*_(ω ∈ [90°, 270°])) and *cis* configurations derived so that $\Delta G_{\mathrm{cis}\to \mathrm{trans}}=-\mathrm{RT}\ln\frac{P_{\mathrm{trans}}}{P_{\mathrm{cis}}}$ at 300 K. To minimize the impact of hysteresis effects, the US simulations were carried in the forward and reverse directions (see Figure S1) and the resulting free energies were averaged.

### Conformational Entropy

The conformational entropies (*S*_conform_) of the solute molecules were calculated with the CENCALC_QUICKSORT software.^35^ Given an MD trajectory, this program discretizes the time evolution of each torsion angle and calculates first its first-order contribution to *S*_conform_. Correlation entropy among the torsional degrees of freedom is taken into account using a reformulated expansion technique termed correlation-consistent multibody local approximation (cc-MLA).^36^ The bias due to finite sampling was minimized by shuffling the elements of the arrays of integer numbers labeling the conformational states populated by the discretized dihedral angles prior to the entropy estimations. By plotting *S*_conform_ as a function of the simulation time, a graphical assessment of the extent of conformational sampling of the corresponding MD trajectory is obtained.^37^

### Quantum Mechanical Calculations

The quantum mechanical (QM) properties of the Ac/NHMe-capped dipeptides were calculated on 100 equally spaced MD snapshots. For the *zwitterionic* Leu-Pro/Leu-Ala dipeptides, 200 snapshots were required to obtain a small statistical uncertainty in the average QM energies comparable to those of the other systems. The coordinates of the solute atoms were first minimized by means of 50 relaxation steps in the presence of a buffer layer of (fixed) OPC waters with a 12 Å thickness. Thus, we performed hybrid quantum mechanical/molecular mechanical (QM/MM) calculations using the QM/MM interface available in the SANDER program.^38^ The QM region, which included all the solute atoms, was described at the HF/cc-pVDZ level of theory^39^ in combination with the third-generation dispersion (D3) correction using the Becke-Johnson damping function.^40^ Subsequently, after having removed the coordinates of the water molecules, single-point HF-D3/cc-pVTZ calculations were carried out on the relaxed structures. In these single-point calculations, solvent effects were taken into account using the SMD parametrization^41^ of the Polarizable Continuum Model (PCM)^42^ that includes both electrostatic and nonelectrostatic (cavity and dispersion) solvation terms, selecting water as the condensed phase. All the QM calculations were performed with the *Gaussian16* program.^43^

To assess the performance of the HF-D3 method in the prediction of *cis–trans* energies, we also calculated the average QM energy of selected systems using the domain-based Local Pair Natural Orbital (DLPNO) coupled cluster method^44–46^ as implemented in the ORCA 5.0 package.^47^ Thus, we performed single-point DLPNO–CCSD­(T)/cc-pVTZ calculations on the QM/MM-relaxed geometries of Ac-Z-NHMe and Ac-Gln-Z-NHMe (Z = Ala or Pro) in the framework of the resolution-of-the-identity approximation (RI) using the appropriate auxiliary basis set and selecting tight DLPNO thresholds for the various truncation parameters.

### IQA Energy Decomposition

In this work, we aim to decompose the isomerization energies of the small peptide molecules into well-defined physical contributions using the Interacting Quantum Atoms approach.^23^ In this respect, the selected level of theory, HF-D3/cc-pVTZ SMD, allows a clear separation of electrostatic, exchange, dispersion and solvation energy contributions.^48^ In what follows, we briefly outline the basic concepts of the original IQA approach and its later extensions that are required to understand our results. Further background on IQA and its applications can be gained from recent reviews.^49–51^

#### Real-Space Energy Decomposition

As shown by the quantum theory of atoms in molecules, atomic regions naturally arise as the attraction basins (Ω_A_) of the gradient field of the electron density. Starting with those atomic basins (Ω_A_), IQA relies on two scalar fields derived from the QM wave function, the first order reduced density matrix ρ_1_(**r**_**1**_ **,r**_**1**_′) and the pair density, ρ_2_(**r**_**1**_,**r**_**2**_). Within the Born–Oppenheimer approximation, IQA decomposes the total energy of a molecular system as a sum of atomic and diatomic energy contributions

1 $$E_{\mathrm{IQA}}^{\mathrm{HF}}=\sum_{A}\left[E_{\mathrm{net}}^{A}+\frac{1}{2}\sum_{B\neq A}E_{\mathrm{int}}^{\mathrm{AB}}\right]$$

where *E*_net_^A^ = *T* ^A^ + *V*_ne_^A^ + *V*_ee_^A^ is the net electronic energy of atom A that includes the kinetic energy *T* ^A^ and the potential energy due to nucleus-electron (ne) attractions and electron–electron repulsions (ee) within Ω_A_. The interaction energy *E*_int_^AB^ = *V*_nn_^AB^ + *V*_ne_^AB^ + *V*_ne_^BA^ + *V*_ee_^AB^ collects various potential energy terms (nn, en, ne and ee) between atoms A and B. The calculation of these energy terms involves the integration of ρ_1_(**r**_**1**_ **,r**_**1**_′) and/or ρ_2_(**r**_**1**_,**r**_**2**_) over the atomic basins Ω_A_ and Ω_B_, what is computationally very expensive due to the numerical evaluation of up to six-dimensional integrals. Moreover, some numerical errors arise in the construction of the interatomic surfaces delimiting the atomic basins and in the radial and angular numerical integrations within those basins.

#### Pairwise Dispersion Effects

As shown in previous work,^48^ the *E*_net_^A^ and *E*_int_^AB^ energies derived from the HF charge density can be readily augmented with the *E*_D3_^AB^ terms that correspond to the *pure* dispersion energy interaction involving atom pairs as calculated with the Grimme’s D3 method^40^

2 $$E_{\mathrm{IQA}}^{\mathrm{HF}‐D3}=\sum_{A}\left[E_{\mathrm{net}}^{A}+\frac{1}{2}\sum_{B\neq A}\left(E_{\mathrm{int}}^{\mathrm{AB}}+E_{\mathrm{D3}}^{\mathrm{AB}}\right)\right]$$

#### Effective Partitioning of Solute–Solvent Effects

Inclusion of solvent effects into IQA depends on the usage of implicit solvent methods like PCM. These methods usually determine the electrostatic reaction-field potential Φ created by the mutual polarization of the solute and the solvent continuum by applying boundary conditions at the solute/continuum interface,^42^ which is normally defined as a solvent excluded surface (SES). The electrostatic contribution to the energy of the solute and the dielectric continuum is expressed as

3 $$E_{solv,elec}=\frac{1}{2}\sum_{I}q_{I}\Phi \left(s_{I}\right)$$

where the *q*_*I*_ terms correspond to a set of point charges assigned to small surface segments (tesserae) located at positions *s_I_* over the solvent excluded surface. The *q*_*I*_ values are obtained by imposing proper boundary conditions on the SES.

The IQA partitioning of total QM energy in solution^26^ relies on the monoelectronic character of Φ given that its decomposition into atomic contributions is straightforward

4 $$\Phi \left(s_{k}\right)=\sum_{A}\Phi^{A}\left(s_{k}\right)$$

so that Φ^A^(**s**_k_) is the electrostatic potential created by the nuclear charge and electron density confined within the atomic basin Ω_A_. This quantity is readily computable within the IQA framework, yielding thus the atomic contribution to the solute–solvent electrostatic energy *E*_solv_

5 $$E_{solv,elec}^{A}=\frac{1}{2}\sum_{k}q_{k}\Phi^{A}\left(s_{k}\right)$$

On the other hand, the SMD version^41^ of the PCM method incorporates various nonpolar solvation terms: cavitation, solute–solvent dispersion and solvent-structural effects, which are accounted for by means of an empirical potential

6 $$E_{\mathrm{solv},\mathrm{CDS}}=\sum_{A}\left(\gamma_{A}+\gamma^{M}\right)\sigma_{A}$$

where γ_A_ and γ^M^ are molecular surface tension parameters and σ_A_ is the solvent-accessible surface of atom A. Hence, the atomic contributions to *E*_solv,CDS_ are combined with the electrostatic *E*_solv,elec_^A^ terms to yield an atomic mapping of the total solvation energy. By adding the resulting terms to the IQA-D3 expression, we perform an energy decomposition analysis of the total HF-D3 SMD energy in the solvent continuum

7 $$E_{\mathrm{IQA}}^{\mathrm{HF}‐D3\;\mathrm{SMD}}=\sum_{A}\left[E_{\mathrm{net}}^{A}+\left(E_{\mathrm{solv},\mathrm{elec}}^{A}+E_{\mathrm{solv},\mathrm{CDS}}^{A}\right)+\frac{1}{2}\sum_{B\neq A}\left(E_{\mathrm{int}}^{\mathrm{AB}}+E_{\mathrm{D3}}^{\mathrm{AB}}\right)\right]$$

#### Separation of Classical (Electrostatic) and Quantum Effects

Further insight into energetic effects can be gained by expressing the reduced pair density matrix as the sum of the Coulombic and exchange-correlation densities *i.e.*, ρ_2_(**r**_**1**_ **,r**_**2**_) = ρ_1_(**r**_**1**_)­ρ_1_(**r**_**2**_) + ρ_2_^xc^(**r**_**1**_ **,r**_**2**_)^23,52^ Thus, we define a classical (electrostatic) component of the pairwise interaction energy, *E*_int,elec_^AB^ = *V*_nn_^AB^ + *V*_ne_^AB^ + *V*_ne_^BA^ + *V*_ee,Coul_^AB^, along with a quantum (exchange-correlation; *V*_xc_^AB^ ≡ *E*_int,xc_^AB^) contribution such that *E*_int_^AB^ = *E*_int,elec_^AB^ + *E*_int,xc_^AB^ It becomes then feasible to assess electronic delocalization (*i.e.*, covalent-like) and purely electrostatic effects in terms of pairwise exchange-correlation (*E*_int,xc_^AB^) and electrostatic energies (*E*_int,elec_^AB^), respectively.^53,54^

The various contributions to the atomic net energy can be organized in a similar fashion so that we distinguish between *quantum* (kinetic and exchange-correlation) and purely electrostatic terms. By rearranging the resulting terms, we obtain additive atomic energies as typically defined in the IQA formalism

8 $$E_{T,\mathrm{xc}}^{A}=T^{A}+V_{\mathrm{ee},\mathrm{xc}}^{A}+\frac{1}{2}\sum_{B\neq A}V_{\mathrm{ee},\mathrm{xc}}^{\mathrm{AB}}$$

9 $$E_{\mathrm{elec}}^{A}=V_{\mathrm{ne}}^{A}+V_{\mathrm{ee},\mathrm{Coul}}^{A}+\frac{1}{2}\sum_{B\neq A}\left(V_{\mathrm{nn}}^{\mathrm{AB}}+V_{\mathrm{ne}}^{\mathrm{AB}}+V_{\mathrm{ne}}^{\mathrm{BA}}+V_{\mathrm{ee},\mathrm{Coul}}^{\mathrm{AB}}\right)$$

10 $$E_{D3}^{A}=\frac{1}{2}\sum_{B\neq A}E_{D3}^{\mathrm{AB}}$$

11 $$E_{\mathrm{solv}}^{A}=E_{\mathrm{solv},\mathrm{elec}}^{A}+E_{\mathrm{solv},\mathrm{CDS}}^{A}$$

In this way, the IQA expression (7) for the HF-D3 SMD energy is reformulated into four separated contributions: quantum (kinetic and exchange-correlation), classical electrostatic, dispersion and solvation

12 $$E_{\mathrm{IQA}}^{\mathrm{HF}‐D3\;\mathrm{SMD}}=\sum_{A}\left(E_{T,\mathrm{xc}}^{A}+E_{\mathrm{elec}}^{A}+E_{\mathrm{solv}}^{A}+E_{D3}^{A}\right)$$

This decomposition can be used to assess the role of electrostatic and quantum effects in intramolecular contacts and/or in chemical bonds. Considering the chemical connectivity through covalent bonds, the energy terms in eq 12 can be also grouped into short-range energies (*e.g*., atomic, 1–2, and 1–3) and medium range energies (1–4 and beyond).

#### IQA Analysis of Isomerization Energy and Steric Energy

From the QM calculations on the MD snapshots, we obtain the average value of the *cis* → *trans* isomerization energy

13 $$\Delta E_{\mathrm{cis}\to \mathrm{trans}}^{\mathrm{HF}‐D3\;\mathrm{SMD}}=\left\langle E_{\mathrm{trans}}^{\mathrm{HF}‐D3\;\mathrm{SMD}}\right\rangle -\left\langle E_{\mathrm{cis}}^{\mathrm{HF}‐D3\;\mathrm{SMD}}\right\rangle$$

which can be also estimated by averaging the IQA-reconstructed energies of each of the MD frames, that is

14 $$\Delta E_{\mathrm{IQA},\mathrm{cis}\to \mathrm{trans}}^{\mathrm{HF}‐D3\;\mathrm{SMD}}=\left\langle E_{\mathrm{IQA},\mathrm{trans}}^{\mathrm{HF}‐D3\;\mathrm{SMD}}\right\rangle -\left\langle E_{\mathrm{IQA},\mathrm{cis}}^{\mathrm{HF}‐D3\;\mathrm{SMD}}\right\rangle$$

The difference between Δ*E*_*cis*→*trans*_^HF‑D3SMD^ and Δ*E*_IQA,*cis*→*trans*_^HF‑D3SMD^ represents a measure of the numerical error inherent in the IQA decomposition of the isomerization energy. Since the isomeric energies are usually quite small (∼1 kcal/mol), it is necessary to average a significant number of IQA-decomposed energies to benefit from numerical error cancellation. For the same reason, a few individual *E*_IQA_^HF‑D3SMD^ energies with numerical errors in the 0.05 quantile were discarded prior to the averaging process.

Using the IQA expression with monatomic and diatomic descriptors (eq 7), the isomerization energy can be decomposed as

15 $$\Delta E_{\mathrm{IQA},\mathrm{cis}\to \mathrm{trans}}^{\mathrm{HF}‐D3\;\mathrm{SMD}}=\Delta E_{\mathrm{net}}+\Delta E_{\mathrm{solv}}+\frac{1}{2}\Delta E_{\mathrm{int}}+\frac{1}{2}\Delta E_{D3}$$

where the contribution derived from the atomic net energies, $\Delta E_{\mathrm{net}}=\sum_{A}\left[{\left\langle E_{\mathrm{net}}^{A}\right\rangle }_{\mathrm{trans}}-{\left\langle E_{\mathrm{net}}^{A}\right\rangle }_{\mathrm{cis}}\right]$ is the atomic *deformation* energy (Δ*E*_net_ ≡ *E*_def_) induced by intramolecular rearrangement. In previous works, the IQA deformation energy has been proposed as a quantitative measure of steric energy.^55^ However, minor perturbations on the electronic distribution can induce large changes in the net atomic energies, what may result in unreliable interpretations of steric effects in terms of *E*_def_. Alternatively, it has been shown^56^ that by removing a charge transfer energy (*E*_CT_) from *E*_def_ the resulting quantity, *E*_ST_ = *E*_def_ – *E*_CT_, can provide a consistent description of steric hindrance in noncovalent complexes and along chemical reaction profiles. In contrast with other IQA descriptors, the value of *E*_ST_ depends on an external reference that is required to estimate *E*_CT_. We adopt the conceptual Density Functional Theory^57^ as a convenient energy reference so that the energy of an isolated atom is expressed as a function of the fractional number of electrons *n*. The *E*(*n*) function can be approximated by a linear piecewise function connecting the consecutive charge states ···, *N* – 1, *N*, *N* + 1, ··· where *N* is the number of electrons of the neutral atom. The corresponding slope and intercept are derived from ionization potential (IP) and electron affinity energies (AE). In our case, we resorted to tabulated values of IP and AE (Table S1) to estimate the average *E*_CT_ of each atomic basin upon *cis–trans* isomerization as *E*_CT_ = *E*(*n_trans_*) – *E*(*n_cis_*).

#### Settings of the IQA Calculations

The IQA decomposition of the QM energies was performed with a modular version of the PROMOLDEN program^58^ that uses localized MOs and employs the multipolar approach^59^ for computing selected interatomic exchange–correlation (xc) energies. The electronic quantities are numerically integrated over finite and irregular integration domains using ultrafine angular and radial grids in atomic spherical quadratures.^60^ A β-sphere around each atom was considered (*i.e*., a sphere completely contained inside the atomic basin), with a radius equal to 60% the distance of its nucleus to the closest bond critical point in the electron density. High-quality Lebedev angular grids were used with 5810 and 974 points outside and within the β-spheres of heavy atoms, respectively (3890 and 590 points for hydrogen atoms). Euler-McLaurin radial quadratures were employed with 512 and 384 radial points outside and inside the β-spheres of heavy atoms, respectively (384 and 256 points for H atoms). The largest value of the radial coordinate in the integrations was 15.0 au for heavy atoms (10.0 au for H atoms). Maximum angular moments, λ_max_, of 10 and 6 were assigned to the Laplace and bipolar expansions of 1/r_12_ outside and within the β-spheres.

The PROMOLDEN code was used to calculate the Bond Critical Point (BCP) properties in the HF/cc-pVTZ SMD charge density ρ­(**r**). The presence of BCPs and the corresponding values of ρ­(**r**) and of the local energy density *H*(**r**) at the BCPs enables one to characterize the presence and strength of polar and nonpolar contacts.^61^

In addition, noncovalent interaction (NCI) plots^62,63^ were calculated for the most populated cluster representatives using the NCIPLOT program and the HF/cc-pVTZ SMD density. The NCI index corresponds to the reduced density gradient, $s\left(r\right)=|\nabla \rho \left(r\right)|/\left(2\sqrt[{3}]{3\pi^{2}}\rho {\left(r\right)}^{4/3}\right)$ , which is a dimensionless measure of the electron density inhomogeneity at point **r**. To elucidate the presence of noncovalent contacts, the analysis of *s*(**r**) is performed by using small isosurfaces (*s*(**r**) ∼ 0.5) and examining both the sign of the second eigenvalue (λ_2_) of the electron density Hessian matrix and the magnitude of ρ­(**r**). In particular, weak noncovalent A···B interactions are identified by *s*(**r**) isosurfaces characterized by negligible electron density overlap (λ_2_ ≤ 0) and low values of ρ­(**r**) (<0.01 au) whereas stronger attractive or repulsive A···B contacts are characterized by λ_2_ < 0 and λ_2_ > 0, respectively, and higher densities (ρ­(**r**) > 0.01 au).

## Results

### Potential of Mean Force and Molecular Dynamics Simulations

#### *cis*–*trans* Energetics

For the Ac-Ala-NHMe and Ac-X-Ala-NHMe systems, our free energy calculations using the US-PMF methodology predict that the *trans* isomeric state is between 2 and 3 kcal/mol more stable than the *cis* state. The free energy profiles also show that the Pro residue stabilizes systematically the *cis* form by about 1 kcal/mol with respect to the X-Ala peptide bonds. Moreover, the *cis–trans* stability is reversed in the *zwitterionic* Leu-Pro molecule (Δ*G*_*cis*→*trans*_ = +0.5 kcal/mol), most likely due to the strength of the intramolecular salt bridge interaction (−COO^–^···^+^H_3_N–, see below), while the *trans* state remains more stable in Leu-Ala (−0.5 kcal/mol).

We also run conventional MD simulations (500 ns) of the *cis* and *trans* forms of the model peptides to sample their conformational motions involving backbone and side chain dihedrals occurring on the ns time scale. To assess the effective amount of sampling, we examined the convergence behavior of the *T*-weighed conformational entropy of the dipeptide molecules. The −*TS*_conform_ plots indicate that at least 100–200 ns are required to properly sample the conformational motions of the dipeptides and that the limiting entropic contributions have very small uncertainties (±0.01–0.04 kcal/mol, see Figure S2), confirming thus that the 500 ns MD simulations performed an extensive conformational sampling. The only exception may correspond to the *cis* state of Ac-Tyr-Pro-NHMe, whose entropy plot has worse convergence properties although the estimated uncertainty of its −*TS*_conform_ value is not large (∼0.1 kcal/mol).

After having extracted 100 equally spaced frames (200 for the Leu-Z *zwitterions*) from each MD trajectory, we calculated the average HF-D3/cc-pVTZ SMD energies of the solute molecules as described in Methods. In addition, we calculated the *cis–trans* average energies in the gas-phase for Ac-Z-NHMe and Ac-Gln-Z-NHMe as obtained by the HF-D3/cc-pVTZ and DLPNO–CCSD­(T)/cc-pVTZ levels. Comparison between the HF-D3 and DLPNO–CCSD­(T) energies shows quite small differences (∼0.1–0.2 kcal/mol in absolute value; see Table S2), supporting thus the adoption of the HF-D3 method to predict the isomeric energies.

The average *cis*–*trans* energies at the HF-D3/cc-pVTZ SMD level are in general close to the US-PMF free energies and qualitatively predict the same *cis*-*trans* preferences, albeit the QM-based energies tend to predict a slightly reinforced Pro *cis* effect. The largest difference between US-PMF and QM energies is found in Ac-Tyr-Pro-NHMe, for which the *trans* state is more stable by nearly 2.0 kcal/mol according to the PMF-US calculations whereas the QM calculations indicate that the *cis* and *trans* states are nearly isoenergetic, the average HF-D3/cc-pVTZ Δ*E* being 0.1 kcal/mol favoring the *cis* form with a standard error of 0.3 kcal/mol.

The US-PMF calculations account for entropy effects arising from the solute and the solvent degrees of freedom. However, the conformational entropy contributions (−*T*Δ*S*_*cis→trans*_^Conform^) obtained from the MD trajectories can give further insight into the role of solute entropy in *cis–trans* isomerization. For the Ac/NHMe capped molecules, the −*T*Δ*S*_*cis→trans*_^Conform^ changes are clearly smaller in absolute value by ∼1–2 kcal/mol than the US-PMF free energy changes and/or the average QM energies, suggesting that the solute enthalpy and/or solvation effects would be the main determinants of their *cis–trans* stability. Nevertheless, it may also be noticed that the *trans* state of the Ac-X-Ala-NHMe molecules seems entropically favored by 0.2–0.5 kcal/mol with respect to that of the Pro-containing dipeptides (see Table 1). For the *zwitterionic* dipeptides, the conformational entropy variations are comparable to the small Δ*G*_*cis→trans*_^US^ values (*e.g*., 0.3 and 0.5 kcal/mol, respectively, for Leu-Pro). However, it cannot be concluded that the *cis–trans* stability of the *zwitterionic* dipetides would be under entropic control because entropy changes due to solute–solvent interactions remain unknown.

**Table 1:** *cis-trans* Energy Components (in kcal/mol) and Change in Dipole Moment (in Debyes) for the Model Peptides Obtained from Classical Simulations and QM Calculations

system	Δ*G*_*cis→trans*_^US‑PMF^ ^a^	*–T*Δ*S*_*cis→trans*_^Conform^ ^b^	Δ*E*_*cis→trans*_^HF‑D3^ ^c^	Δμ_*cis→trans*_ ^d^
Ac-Pro-NHMe	–1.4 ± 0.2	0.01 ± 0.02	–0.8 (0.1)	–2.3 (0.3)
Ac-Ala-NHMe	–2.1 ± 0.1	0.11 ± 0.01	–2.4 (0.2)	0.1 (0.5)
Ac-Gln-Pro-NHMe	–1.6 ± 0.3	0.01 ± 0.04	–1.0 (0.3)	–1.9 (0.4)
Ac-Gln-Ala-NHMe	–2.7 ± 0.1	–0.58 ± 0.03	–2.3 (0.3)	0.2 (0.6)
Ac-Leu-Pro-NHMe	–1.9 ± 0.6	0.18 ± 0.02	–0.7 (0.3)	–3.8 (0.4)
Ac-Leu-Ala-NHMe	–2.9 ± 0.0	–0.55 ± 0.01	–2.0(0.3)	1.4 (0.5)
Ac-Tyr-Pro-NHMe	–2.0 ± 0.2	–0.26 ± 0.09	0.1 (0.3)	–1.7 (0.4)
Ac-Tyr-Ala-NHMe	–2.9 ± 0.2	–0.55 ± 0.01	–2.9 (0.3)	0.6 (0.4)
Leu-Pro	0.5 ± 0.2	0.30 ± 0.01	0.9 (0.2)	
Leu-Ala	–0.5 ± 0.1	–0.35 ± 0.01	–0.4 (0.2)	

a Average value of the forward and backward US-PMF simulations.

b Difference in the *T*-weighed conformational entropy of the solute molecules (average and standard deviation as obtained from the MD simulations).

c *cis–trans* energy obtained from average HF-D3/cc-pVTZ SMD energies Δ*E*_*cis→trans*_^HF‑D3^ = ⟨*E*_*trans*_^HF‑D3^⟩ – ⟨*E*_*cis*_^HF‑D3^⟩ (standard error in parentheses).

d *cis–trans* change in the average HF-D3/cc-pVTZ SMD dipole moment (standard error in parentheses).

#### Structural Analysis

To characterize the conformational motions of the peptide molecules during the MD simulations, we examined first the puckering of the prolyl ring that flips between the Cγ­(*endo*) and Cγ­(*exo*) conformations with a frequency of ∼0.08 ps^–1^. According to the ff19SB-OPC force field, the Cγ­(*endo*) pucker is dominant for the Ac/NHMe-capped systems with an abundance between 66 and 71% in the *cis* isomers and 58–62% in the *trans* ones (see Table S3). For the *zwitterionic* Leu-Z dipeptides, both puckers are more evenly populated, the Cγ­(*endo*) abundances being 56 and 46% in the *cis* and *trans* forms, respectively. Overall, the simulations indicate that the Cγ­(*endo*) pucker is more likely in the *cis* isomers, what is in line with X-ray data^64^ showing that *cis*-Pro residues in protein structures adopt preferentially the Cγ­(*endo*) pucker (81%) while the Cγ­(*endo*) and Cγ­(*exo*) conformations tend to be equally populated in *trans*-Pro. A higher population of Cγ­(*endo*) conformers (∼81%) has been also observed in NMR studies^65^ in solution for the Gly-Pro-Gly-Gly and Val-Ala-Pro-Gly tetrapeptides.

The backbone conformations were characterized by computing the Ramachandran plots of the residue-averaged (φ,ψ) dihedral angles (Figure S3). In general, most of the backbone torsions lie within the −90° < φ < −40° and 120° < ψ < 180° intervals, which would be compatible with a PPII helix motif in polypeptide structures. The backbone angles also populate other (φ,ψ) regions that are typical of extended β-structures and compact helical forms, which become more abundant (∼10–25%) in the Ala-containing peptides. Further details are revealed by the clustering of the coordinates of the heavy atoms. Inspection of the most representative conformers (Figure 1) reveals that (i) the flexibility of the backbone chain is moderate, the Pro-containing peptides being more rigid than their Ala-containing counterparts as suggested by the Ramachandran plots and the entropy calculations, (ii) the *cis*-forms are more compact (as confirmed by molecular surface area and radius of gyration calculations; see Table S4), and (iii) the Gln, Leu and Tyr side chains adopt multiple conformations.

**Figure 1:**
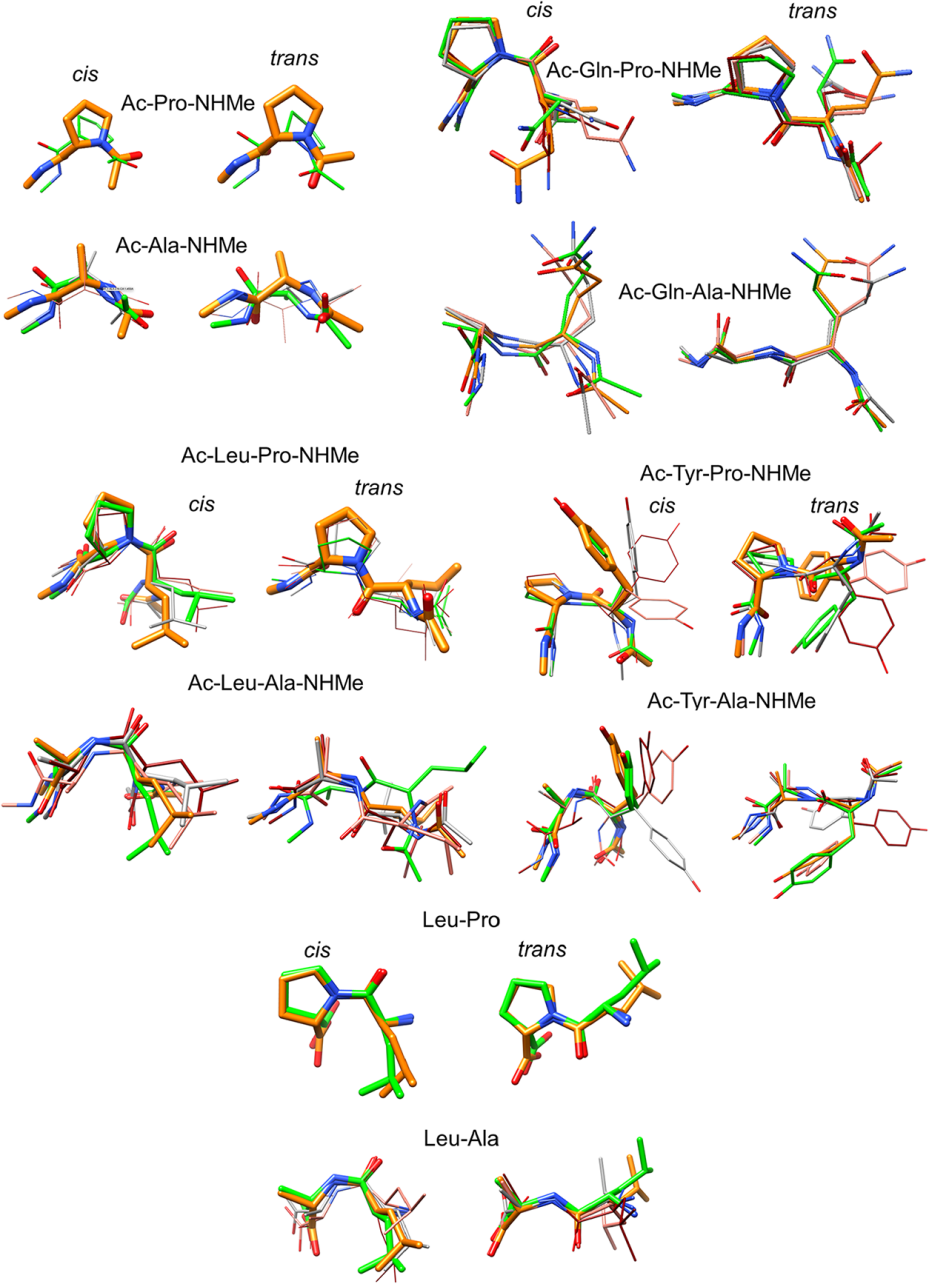
Superposition of top cluster representatives (stick thickness is proportional to cluster abundance) of the peptide conformations explored by the MD simulations.

### Assessing Noncovalent Intramolecular Interactions

To better determine the relationship between *cis*/*trans* states and intramolecular contacts, we analyzed the HF charge density (ρ) of the short peptides by locating the corresponding BCPs associated to noncovalent interactions and using the solute coordinates extracted from the MD simulations. In this way, interatomic contacts were first characterized in terms of quantum topology descriptors in line with the real space IQA energy partitioning.

The abundance and average electronic properties of the BCPs of interest are collected in Table S5. These BCPs, which represent donor–acceptor interactions between closed-shell systems (*i.e*., small value of ρ­(r_c_) ∼0.01 e^–^ and a small and positive value of the Laplacian ∇^2^ρ­(r_c_) ∼ 0.04 au),^61^ pinpoint not only conventional (NH···O/OH···O) and (CH···O) hydrogen bonds, but also other favorable interactions involving C­(π)···N/O or C­(π)···HC contacts^66^ as those present in the Ac-Tyr-Z-NHMe system. Similarly, the presence of BCPs for unusual CH···HC contacts, which are normally associated to attractive exchange-correlation IQA interaction energies,^67^ may be seen as evidencing steric proximity between nonpolar moieties like −CαH– or −CδH_2_– at the X and Pro residues.

Table 2 summarizes the number and abundance of the noncovalent BCPs. The *cis* isomers give rise to ∼1.5–2-fold more intramolecular contacts than the *trans* isomers and, in addition, they tend to be more stable. More specifically, the *cis* configuration is compatible with Ac@C=O···NH@NHMe and Ac@C=O···_3_HC@NHMe contacts, which are lost upon *cis* → *trans* rearrangement, and is more adequate for CH···HC contacts. We also found that the Pro-containing peptides are prone to exhibit more BCPs than their Ala-containing counterparts involving various side chain···backbone atom pairs as well as the −CH_2_– groups of the prolyl ring.

**Table 2:** Average Number of Noncovalent BCPs (*n*_con_) in the HF/cc-pVTZ SMD Charge Density with Population Above 5%^a^

Ac-Z-NHMe			Ac-Gln-Z-NHMe		Ac-Leu-Z-NHMe	
	Z = Pro	Z = Ala	Z = Pro	Z = Ala	Z = Pro	Z = Ala
*n*_con_	4/1	2/1	23/15	16/7	21/9	16/6
*p*_aver_	0.16/0.31	0.06/0.05	0.14/0.16	0.14/0.09	0.15/0.19	0.13/0.09
*p*_max_	0.35/0.31	0.06/0.05	0.36/0.35	0.48/0.17	0.43/0.35	0.38/0.21

	Ac-Tyr-Z-NHMe		Leu-Z	
	Z = Pro	Z = Ala	Z = Pro	Z = Ala
*n*_con_	18/19	18/9	21/11	9/0
*p*_aver_	0.18/0.14	0.13/0.07	0.09/0.13	0.15/-
*p*_max_	0.43/0.38	0.60/0.10	0.28/0.45	0.34/-

a The average and maximum fractional population (*p*_aver_ and *p*_max_) are also indicated. All values are given for the *cis* (left)/*trans* (right) isomers.

The case of the Ac-Tyr-Z-NHMe (Z = Pro, Ala) peptides may deserve particular attention. On the one hand, the BCP analysis confirms that their *cis* isomers exhibit C­(π)···HC interactions involving the Tyr side chain and the pyrrolidine/methyl CH bonds of Pro/Ala. On the other hand, in the *trans* state of Ac-Tyr-Pro-NHMe, some of those C­(π)···HC contacts are replaced by Tyr@C­(π)­H···O@Pro and Pro@CδH···O@Ac H-bonds so that the *cis* and *trans* isomers give a similar number of contacts (see Tables 2 and S5).

For the *zwitterionic* Leu-Z dipeptides (Z = Pro, Ala), the BCP analysis characterizes the presence of a monodentate salt bridge contact between the terminal ammonium and carboxylate groups that is only feasible in the *cis* isomeric state. It is also suggested that the −COO^–^···^+^H_3_N– contact would be slightly stronger in Leu-Pro in terms of the abundance, bond distance and charge accumulation of the corresponding BCPs.

Although the average BCP properties assess the relative importance of specific contacts, the topological properties of ρ fluctuate in response to small changes of molecular geometry and cannot provide an exhaustive description of all noncovalent or through-space interactions. To complement the BCP analysis, we calculated several NCI plots in terms of the reduced density gradient *s*(**r**) as described in the Methods section. The qualitative analysis of the *s*(**r**) isosurfaces ascertains the interatomic contact regions that are associated with noncovalent interactions. This analysis was only performed for the representative structures of the most populated clusters and a few selected examples are shown in Figure 2 (see also Figure S4). One the one hand, the NCI plots confirm the main intramolecular contacts detected by the BCP analysis (*i.e*., CH···O, CH···C­(π) and CH···HC), but give also further details about their extension and localization. Thus, the polar A···B interactions give rise to small isosurface (*s*(**r**) = 0.5) patches located around the A···B midpoint while the nonpolar ones (*e.g.*, CH···C­(π) and CH···HC) are represented by larger isosurfaces denoting less specific and nondirectional interactions. The total area of the reduced density gradient isosurfaces suggests again that the *cis* isomer gives closer intramolecular contacts (see Figures 2 and S4). More interestingly, we found that the NCI plots detect one additional intramolecular contact in the *trans* state, which does not give rise to a BCP in the charge density. It corresponds to an isosurface patch placed in between the carbonylic O atom of the Ac/X-Z peptide bond and the subsequent backbone C=O group. Such contact can be tentatively ascribed to the *n* → π* electronic delocalization between nearby amide bonds that has been proposed to explain the conformation of peptide chains (see below).

**Figure 2:**
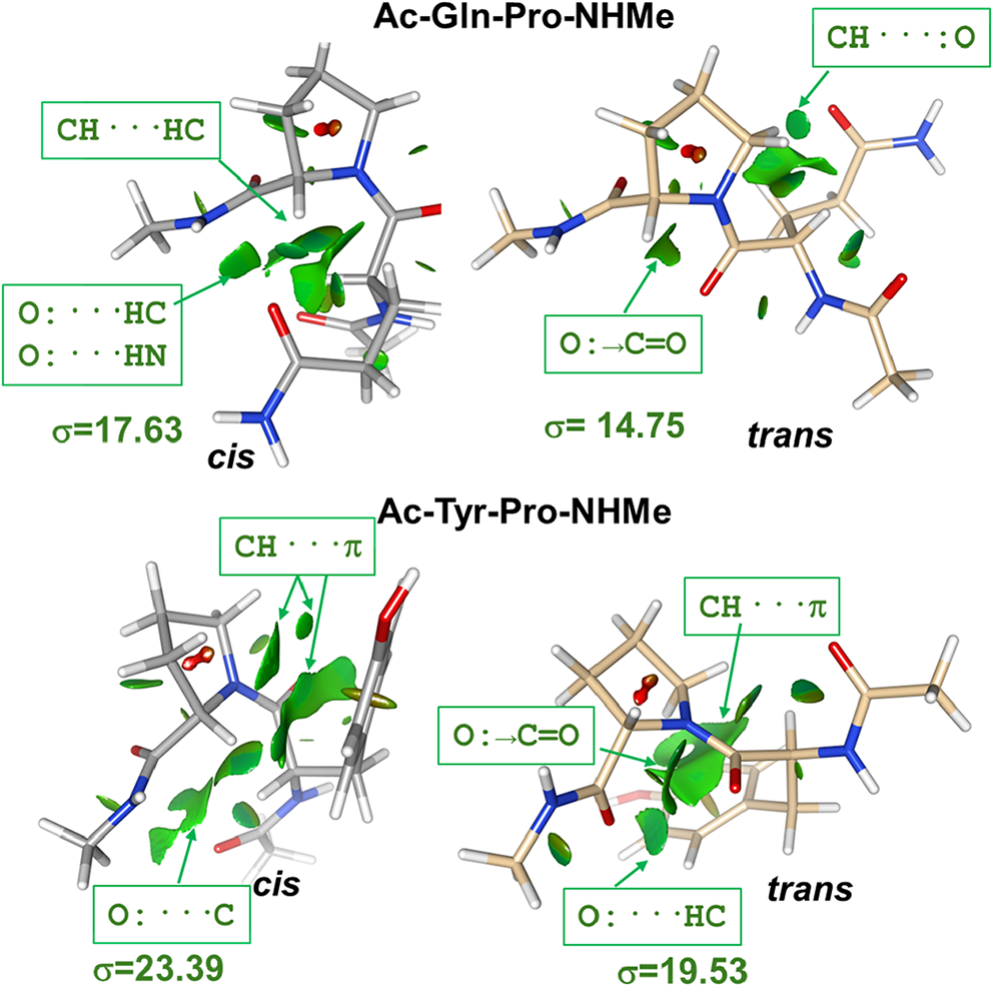
Images of NCI plots (*s* = 0.5) for the most populated cluster representatives of the Ac-X-Pro-NHMe (X = Gln, Tyr) peptides with the green regions indicating noncovalent interactions (main contacts are identified). Surface area of the isosurface elements (σ in Å^2^) are also indicated.

### IQA Energy Decomposition Analysis

To incorporate the conformational properties of the dipeptide molecules in our QM energetic analysis of the Pro *cis* effect, we focused on the IQA decomposition of the conformationally averaged *cis–trans* energy differences (Δ*E*) as defined in the Methods section (eq 12). Thus, Table 3 summarizes the IQA contributions to the isomerization energy at the HF-D3/cc-pVTZ SMD level of theory. Other IQA steric and electronic energy descriptors are presented in Table 4. To enhance the visual interpretation of the energy decomposition analysis, Figure 3 displays the structures of cluster representatives of the small peptides onto which the numerical values of the atomic IQA quantities are mapped. In this way, the relevance of specific atoms for the *cis–trans* energy variations can be more easily grasped.

**Table 3:** Average IQA Energy Components (Kinetic and Exchange–Correlation, Δ*E*_T,xc_, Electrostatic, Δ*E*_elec_, Solvation, Δ*E*_solv_ and Dispersion, Δ*E*_D3_) for *cis*-*trans* Isomerization (in kcal/mol)

	Ac-Z-NHMe		Ac-Gln-Z-NHMe		Ac-Leu-Z-NHMe		Ac-Tyr-Z-NHMe		Leu-Z	
	Z = Pro	Z = Ala	Z = Pro	Z = Ala	Z = Pro	Z = Ala	Z = Pro	Z = Ala	Z = Pro	Z = Ala
Δ*E*_T,xc_	6.1	1.2	–2.9	–7.5	–0.9	–7.5	1.9	2.4	–9.5	–7.8
Δ*E*_elec_	–8.9	–5.3	–1.6	4.4	–4.1	4.4	–4.9	–6.4	46.4	33.7
Δ*E*_T,xc_ + Δ*E*_elec_	–2.8	–4.1	–4.5	–3.2	–5.0	–3.1	–3.8	–4.0	37.0	25.9
Δ*E*_D3_	0.6	1.2	2.1	3.0	2.5	3.0	1.6	2.1	2.2	3.2
Δ*E*_solv_	1.3	0.4	0.8	–1.6	1.3	–2.1	1.3	–1.5	–38.5	–29.4
Δ*E*_IQA_ (Δ*E*)^a^	–1.5 (−1.4)	–3.7 (−3.6)	–3.6 (−3.1)	–4.8 (−5.3)	–3.7 (−3.2)	–5.2 (−5.0)	–1.7 (−1.5)	–5.5 (−5.0)	–1.5 (−1.3)	–3.6 (−3.6)

a Isomerization energy reconstructed from the IQA terms­(*i.e*., Δ*E*_IQA_ = Δ*E*_T,xc_ + Δ*E*_elec_ + Δ*E*_solv_; excluding dispersion). The Δ*E* values in parentheses correspond to the equivalent isomerization energy derived from the HF/cc-pVTZ wave function.

**Table 4:** Average Values (in kcal/mol) of the *cis* → *trans* IQA Steric Energy (*E*_ST_) Descriptors and of the Pairwise Exchange–Correlation Energies (Δ*E*_int,xc_) Involving the Carbonyl Groups of the X-Pro/X-Ala Residues

	Ac-Z-NHMe		Ac-Gln-Z-NHMe		Ac-Leu-Z-NHMe		Ac-Tyr-Z-NHMe		Leu-Z	
	Z = Pro	Z = Ala	Z = Pro	Z = Ala	Z = Pro	Z = Ala	Z = Pro	Z = Ala	Z = Pro	Z = Ala
global *E*_ST_	4.2	3.3	–3.4	–5.3	–5.4	–6.7	–6.2	0.4	–16.3	–10.0
*E*_ST_(Cα(Ac/X))^a^	–3.7	–3.4	–1.0	–2.8	–0.2	–6.1	–3.5	–4.1	–0.3	–2.8
*E*_ST_(Cα(Z))	0.6	–0.6	–0.6	–1.8	–2.4	–2.2	–3.8	–4.0	–2.2	–4.7
*E*_ST_(Cδ(Pro))	1.1		1.5		4.2		5.5		3.0	
Δ*E*_int,xc_(Ac/X@C=O, Z@C=O)	–3.7	–2.0	–3.1	–2.0	–3.0	–2.0	–2.2	–1.6	–1.6	–1.0
Δ*E*_int,xc_(Ac/X@O, Z@C=O)	–3.7	–2.0	–3.2	–2.0	–3.1	–1.9	–2.3	–1.6	–1.8	–1.2
Δ*E*_int,xc_(Ac/X@O, Z@C)	–1.9	–1.0	–1.7	–1.1	–1.6	–1.1	–1.4	–0.9	–1.0	–0.5

a Including CH_3_– for Ac and –CαH– for X = Gln, Leu, Tyr.

**Figure 3:**
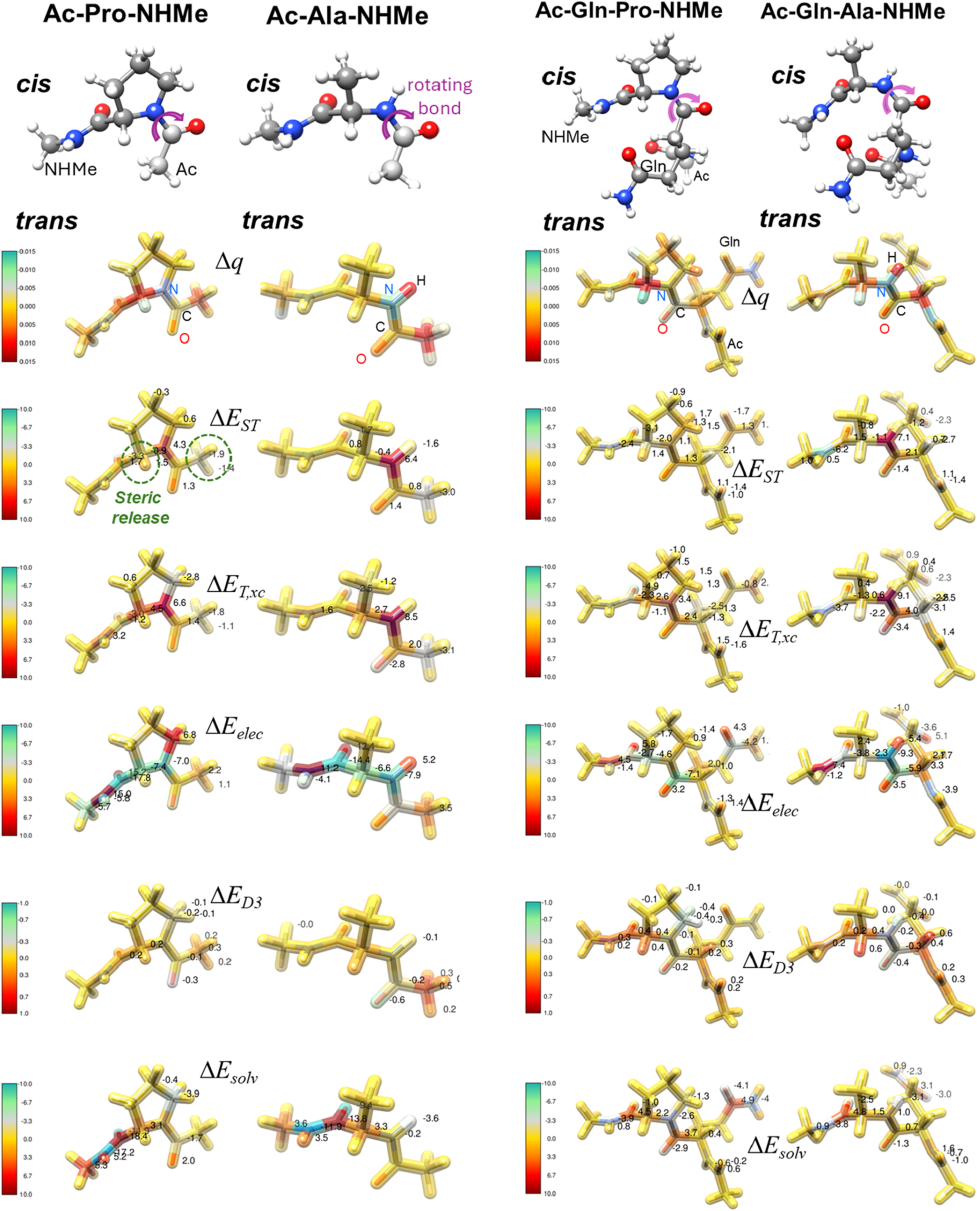

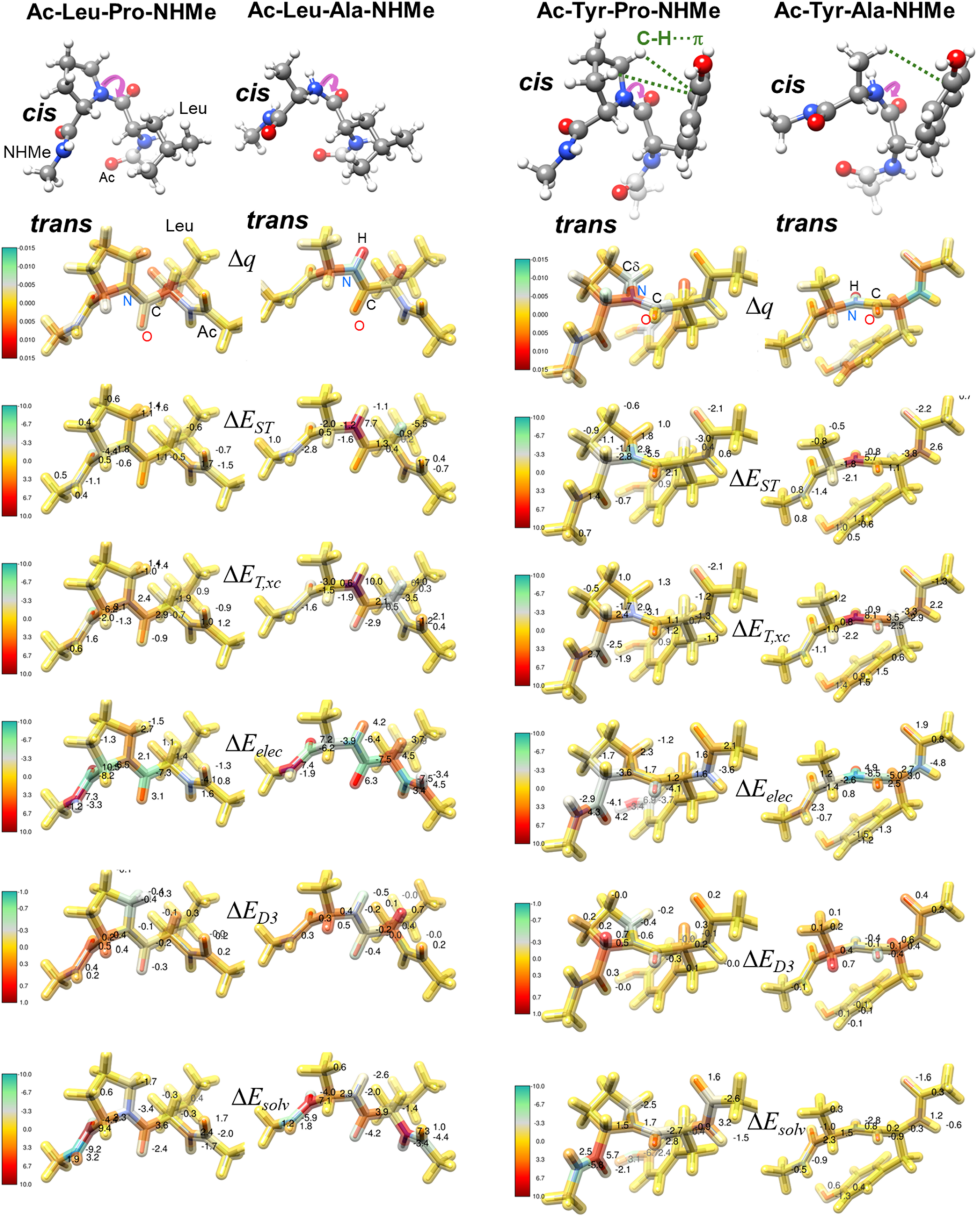

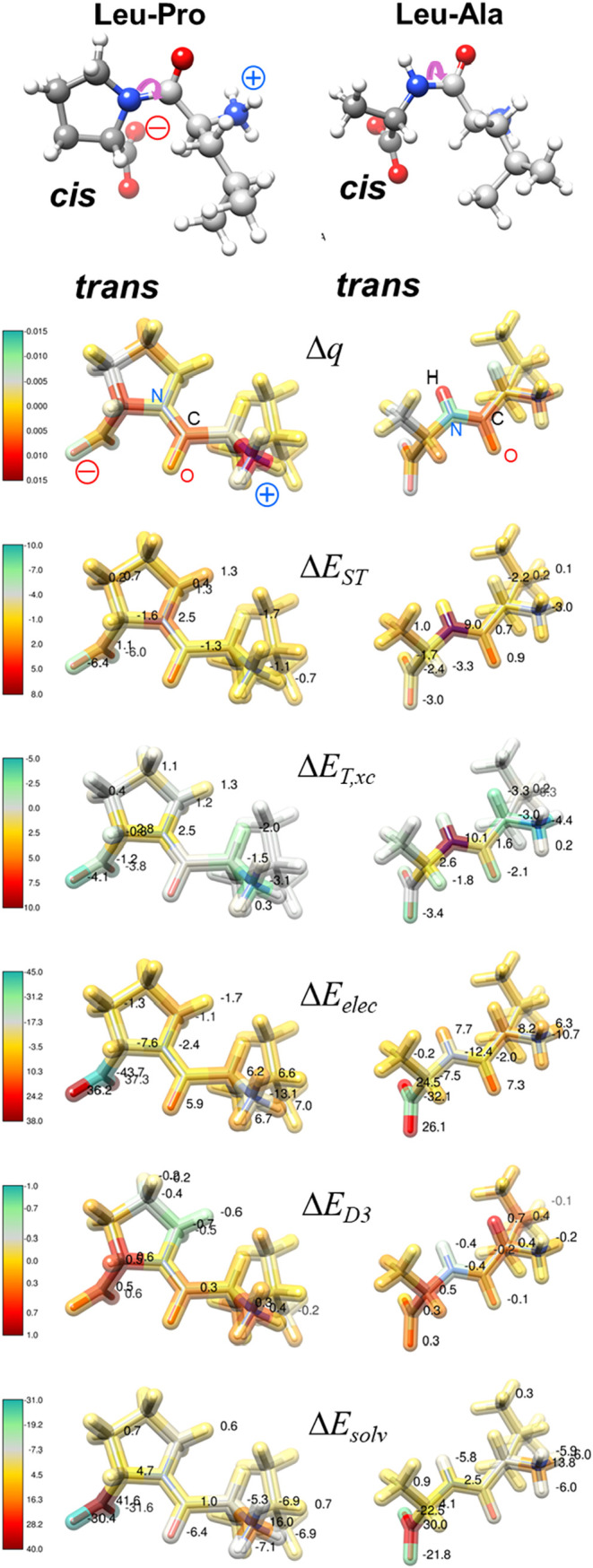
Most important cluster representatives of the *cis* (ball-and-stick) and *trans* (stick) isomers of the model peptides. Multiple views of each *trans* structure are displayed embedded within thicker stick models rendered as colored and partially translucent objects. The coloring patterns represent the atomic changes upon the *cis* → *trans* rearrangement that arise in the QTAIM charges (Δ*q* in e^–^) and in the various IQA atomic energy terms (steric, Δ*E*_ST_; kinetic and exchange-correlation, Δ*E*_T,xc_; electrostatic, Δ*E*_elec_; dispersion, Δ*E*_D3_; and solvation, Δ*E*_solv_). Note that, as defined in eq 12, the total Δ*E*_*cis*→*trans*_ is given by Δ*E*_T,xc_ + Δ*E*_elec_ + Δ*E*_D3_ + Δ*E*_solv_ while Δ*E*_ST_ is a separate energetic descriptor derived from atomic deformation energies (see text for further details). In the colored *trans* stick models, the color of each half-bond matches that of the attached atom. For the sake of comparison, the IQA atomic energy changes in the Ac/NHMe capped peptides are represented with the same color map ranging from −10.0 kcal/mol (green, favoring the *trans* state) to 10.0 kcal/mol (red, favoring the *cis* form). For the *zwitterionic* Leu-Z, energy variations occur in wider and specific ranges. The most important electronic (in e^–^) and energy changes (in kcal/mol) at the atomic level are indicated.

To estimate the magnitude of the numerical errors in the IQA quantities, we compared the IQA-reconstructed energy Δ*E*_IQA_ (excluding dispersion) with the equivalent term derived from the analytical integration of the HF wave function (Δ*E*). The observed differences, ∼0.0–0.6 kcal/mol in absolute value, indicate that numerical errors are relatively small and comparable to the statistical uncertainty of the average *cis–trans* energy difference (∼0.2–0.3 kcal/mol; see Table 1). Since most of the IQA contributions collected in Table 3 change significantly upon isomerization by a few kcal/mol, the qualitative trends outlined from the energy decomposition analysis should be reliable regardless of the residual numerical errors.

#### Steric Descriptors

As explained in Methods, elusive steric energy can be associated with the change of the net atomic energies with respect to a suitable reference provided that charge-transfer effects are effectively removed. However, several atoms undergo a significant electronic rearrangement ongoing from the *cis* to *trans* isomer such as those of the X-Pro/X-Ala amide bonds and those involved in the rupture/formation of H-bond interactions. Consequently, the global steric energy (*E*_ST_ in Table 4) cannot be ascribed to reflect only the mitigation of steric repulsions around the central peptide bond. For example, the positive *E*_ST_ values (>3 kcal/mol) for the *cis* → *trans* process of Ac-Z-NHMe would be indicative of more steric hindrance in the *trans* isomer, what is in contrast with expectations. In fact, the largest *E*_ST_ contributions correspond to the NH and C=O moieties of Ac-Z-NHMe (see Figure 3) and, since these groups are essentially unaffected by steric repulsions during the *cis* → *trans* rearrangement, it turns out that the net atomic energies embodied within *E*_ST_ can be substantially affected by other electronic/electrostatic effects regardless of the *ad hoc* charge-transfer correction.

Perhaps of more interest can be the examination of the specific atomic contributions to *E*_ST_ that are commonly assumed to be involved in the Pro *cis* effect, that is, the −CαH– positions of the X-Z amino acids that can be in clashing proximity in the *cis* isomer so that, upon conversion to the *trans* isomer, a certain steric strain release would occur (see Scheme 2a). In the X-Pro systems, the strain release of the −CαH– groups would be partially compensated by the CδH_2_ group that is expected to exert a similar steric repulsion in the *trans* state. Such assumptions are partly supported by the fragment *E*_ST_ contributions collected in Table 4. Thus, both the *E*_ST_(Cα­(Ac/X)) and *E*_ST_(Cα­(Z)) values are negative for nearly all the examined *cis* → *trans* transitions (the only exception is *E*_ST_(Cα­(Z)) in Ac-Pro-NHMe), what seems in consonance with the expected release of steric strain, while the equivalent *E*_ST_(Cδ­(Pro)) terms are positive suggesting a certain accumulation of steric strain in the *trans* state. However, the magnitude and relative values of these descriptors do not allow us to assign the Pro *cis* effect to purely steric repulsion. For example, *E*_ST_(Cα­(X)) has a small value (−0.23 kcal/mol) in the Ac-Leu-Pro-NHMe peptide while the equivalent term in Ac-Leu-Ala-NHMe is more negative (−6.10 kcal/mol) but inspection of the most likely conformers in Figure 1 and the BCP properties in Table S5 shows that the –CαH– group gives comparable contacts both in *cis* Ac-Leu-Ala-NHMe and in *cis* Ac-Leu-Pro-NHMe, in contrast with their dissimilar *E*_ST_(Cα­(X)) indexes. Moreover, besides the selected steric quantities in Table 4, many other atomic positions present comparable *E*_ST_ contributions as shown in Figure 3 and the *cis* → *trans* transformation is accompanied by notable changes in the different energy components that cannot be ignored to explain the Pro *cis* effect.

**Scheme 2:**
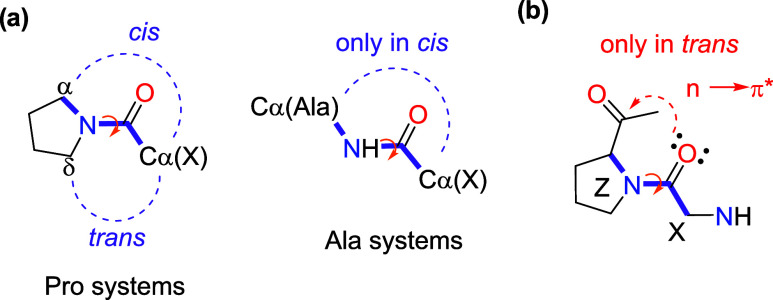
(a) Steric Repulsions between C α/Cδ Sites; (b) Electronic Hyperconjugation Effect Involved in *trans* Isomers

#### Assessment of Hyperconjugation Effects

It has been formerly noticed^53^ that the interatomic exchange-correlation energies (*E*_int,xc_^AB^) can be used to assess primary bond interactions (*e.g*., covalent bonds) as well as secondary stereoelectronic effects. In this way, the energetic impact of the *n* → π* electronic delocalization between an electron lone pair of the O atom at the Ac/X-Z peptide bond and the subsequent backbone carbonyl group (see Scheme 2b and the NCI plots in Figure 2) can be correlated with their contributions to the pairwise *E*_int,xc_ energies. Hence, we evaluated the *cis–trans* Δ*E*_int,xc_ difference for the (O, C) pair of atoms, but also for the donor O atom and the acceptor C=O and the two C=O groups (see Table 4). The same trend is obtained from these three Δ*E*_int,xc_ descriptors, which have negative values ranging within ∼(−4, −1) kcal/mol. Note that the Δ*E*_int,xc_ (Ac/X@C=O, Z@C=O) and Δ*E*_int,*xc*_(Ac/X@O, Z@C=O) values are nearly identical whereas Δ*E*_int,*xc*_(Ac/X@O, Z@C=O) is about 1 kcal/mol below Δ*E*_int,xc_(Ac/X@O, Z@C), what is in agreement with the *n* → π* orbital description. We also confirmed that the *cis* isomers present *E*_int,xc_ values for the *n* → π* interaction that are negligible (data not shown for brevity) so that the relative Δ*E*_int,xc_ values reflect the specific *trans* stabilization effect. All the Δ*E*_int,xc_ descriptors in Table 4 are about 1–2 kcal/mol more negative when Z = Pro, suggesting thus that the Pro-containing peptides would favor the *n* → π* interaction with respect to their Ala-containing counterparts, what is in agreement with former proposals.^5^ Therefore, it can be concluded that the IQA-based Δ*E*_int,xc_ terms capture the energetic impact of the *n* → π* hyperconjugation.

Although the presence of the *n* → π* interaction in the *trans* isomers is consistent with our IQA analysis, it cannot rationalize the observed Pro *cis* effect in Ac-X-Pro-NHMe. This is so because the *n* → π* electronic delocalization would favor a Pro *trans* effect, but also because of the importance of other energetic contributions associated to the backbone carbonyl groups. For example, the alignment of the C=O bonds in the *trans* configuration is accompanied by stronger electrostatic attractions (*e.g*., Δ*E*_int,elec_(Ac/X@C=O, Z@C=O) amounts to −14.80 and −10.81 kcal/mol for Ac-Gln-Z-NHMe with Z = Pro, Ala), but worse solvation properties (*e.g*., Δ*E*_solv_(Ac/X@C=O, Z@C=O) = +4.27 and +1.83 for Ac-Gln-Z-NHMe with Z = Pro, Ala).

Other hyperconjugation effects such as those presumably involved in C­(π)···HC interactions can be also assessed by examining the relevant exchange-correlation interaction energies. For example, the average Δ*E*_int,xc_ values reflecting the *cis* → *trans* modification in the Tyr@C­(π)···HC interactions amount to +2.20 and +2.90 kcal/mol for Ac-Tyr-Pro-NHMe and Ac-Tyr-Ala-NHMe, respectively (considering the – CβH_2_–/–CγH_2_– and −CβH_3_ groups of Pro and Ala), suggesting thus that the C­(π)···HC contacts in the *cis* state might be more effective in the Ala peptide. Nevertheless, the formation/rupture of C­(π)···HC contacts implies further energetic variations (*e.g*., a concomitant loss of dispersion attractions of 0.73 and 0.82 kcal/mol) and other contacts can have a comparable energetic impact. Therefore, it is necessary to complement the energetic assessment of any localized and specific (steric or electronic) effects with a broader energetic analysis to better understand the Pro *cis* effect.

#### Total Energy Partitioning into Physical Components

In the Methods section, we introduced the IQA protocol (eqs 12–15) for partitioning the *cis–trans* isomerization energy (Δ*E*) into four separated contributions: quantum (kinetic and exchange-correlation), electrostatic, dispersion and solvation, which can be further partitioned into effective atomic quantities. This proposed decomposition is suitable to assess the role of energy contributions on intramolecular contacts and/or in chemical bonds.

From our decomposition analysis of the total Δ*E* energies (see Table 3), we note first that the sign and magnitude of the changes in the classical (*i.e*., electrostatic Δ*E*_elec_) and quantum (*i.e*., kinetic and exchange-correlation Δ*E*_T,xc_) components depend on the identity and properties of the residues around the rotating amide bond. For instance, Δ*E*_T,xc_ > 0 in the Ac-Z-NHMe (Z = Pro, Ala) and Ac-Tyr-Z-NHMe systems, but Δ*E*_T,xc_ < 0 for the *cis* → *trans* conversion of Ac-X-Z-NHMe (X = Gln, Leu). The electrostatic component favors the *trans* isomer of the Pro-containing systems Δ*E*_elec_ < 0, what is in consonance with the *cis* → *trans* reduction in their average dipole moment (see Table 1). The overall balance of electrostatic and kinetic-*xc* terms favors the *trans* isomer by a few kcal/mol (*i.e*., Δ*E*_elec_ + Δ*E*_T,xc_ < 0), what may be partly due to the enhanced C=O:···C=O interactions in the *trans* state, but also to other electronic deformation (intraatomic) and pairwise interaction effects. The *trans* preference shown by Δ*E*_elec_ + Δ*E*_T,xc_ does not justify the Pro *cis* effect observed in all the peptide models. While Δ*E*_elec_ + Δ*E*_T,xc_ is about 1.3 kcal/mol less negative for Ac-Pro-NHMe than for Ac-Ala-NHMe, what would be in line with the Pro *cis* effect (*i.e*., | Δ*E* (Ac-Pro-NHMe) | < | Δ*E* (Ac-Ala-NHMe) |), such trend is reversed for the Ac-Gln-Z-NHMe and Ac-Leu-Z-NHMe peptides.

Another energetic feature revealed by our IQA partitioning is the loss of attractive dispersion energy upon the *cis* → *trans* isomerization leading to less compact conformations, as all the corresponding Δ*E*_D3_ values in Table 3 are positive, what agrees well with the reduction of intramolecular contacts in the *trans* state as characterized by the BCP/NCIPLOT data. The Pro-containing peptides have Δ*E*_D3_ values which are ∼0.5–1 kcal/mol lower than those of the Ala-containing peptides, showing thus that the −CγH_2_–CδH_2_– moiety of the pyrrolidine ring reduces the dispersion energy difference between the two isomers. As a result, this energetic effect promotes the *trans* isomer of the Pro-containing peptides with respect to their Ala-containing counterparts.

Table 3 shows that the role of solute–solvent interactions on the *cis–trans* equilibrium is quite notable and systematic for the Ac-Z-NHMe and Ac-X-Z-NHMe systems. Thus, the SMD solvent model promotes the *trans* form of Ac-X-Ala-NHMe (Δ*E*_solv_ < 0), which has a higher polarity than the *cis* form (Δμ > 0; see Table 1), while the opposite trend results for Ac-X-Pro-NHMe (Δ*E*_solv_ > 0 and Δμ < 0). Although the corresponding Δ*E*_solv_ values for the Ac-Pro-NHMe and Ac-Ala-NHMe pair are positive, 1.3 and 0.4 kcal/mol, respectively, they represent a larger stabilization (∼0.9 kcal/mol) of the *cis* isomer of Ac-Pro-NHMe than that of Ac-Ala-NHMe. Consequently, solvent effects tend to stabilize the *cis* state (>1.0 kcal/mol) of the Pro-containing molecules with respect to the peptide molecules containing the Ala residue. Therefore, it turns out that the intramolecular IQA decomposition underlines the role played by solvation to control the Pro *cis* effect in the capped dipeptides.

As expected, the presence of charged groups in the vicinity of the X-Pro/X-Ala linkages can have a marked influence on the *cis–trans* equilibrium. Thus, the partitioning of Δ*E* for the *zwitterionic* Leu-Z molecules (Z = Pro, Ala) is dominated by the presence of carboxylate and ammonium groups so that, in absolute value, the electrostatic and solvent contributions to Δ*E* have large values ranging between 29 and 46 kcal/mol. Concerning the relative stability of the *cis*-*trans* states, the kinetic and exchange energy favors again the trans *form* (*i.e*, Δ*E*_T,xc_ < 0) while the loss of electrostatic energy due to the loss of the −COO^–^···^+^H_3_N– salt bridge (Δ*E*_elec_ > 0) is compensated by more intense solvation (Δ*E*_solv._ < 0). As shown in Figure 1, the salt-bridge makes the *cis* Leu-Z systems more rigid and more tightly packed so that adoption of the *trans* isomeric state results in a significant loss of dispersion attraction in Leu-Pro and Leu-Ala (Δ*E*_D3_ > 2 kcal/mol). Overall, as above-mentioned, the *cis* isomer of Leu-Pro is 0.9 kcal/mol more stable whereas Leu-Ala prefers the *trans* isomer by 0.4 kcal/mol (see Table 1). As solute–solvent interactions stabilize the *trans* configuration of Leu-Pro and Leu-Ala (in contrast with the capped peptide models), the *cis* stabilization in Leu-Pro may result from a stronger −COO^–^···^+^H_3_N– contact as suggested by the BCP analysis and the greater electrostatic penalty involved in its rupture (see Table 3).

#### Atomic and Fragment Distribution of Energetic Components

In principle, the atomic decomposition achieved by eq 15 can give further insight into the *cis–trans* energy differences albeit the high number of IQA terms complicates the analysis tasks. By examining the molecular models in Figure 3, we observe first minor changes (±0.01 e^–^) in the electronic population of a few atomic basins that mainly affect to the rotating amide bond, but also to the H and N/O atoms that form/break noncovalent contacts upon the *cis–trans* rearrangement. More specifically, the largest electronic rearrangement in the Ac-Z-NHMe/Ac-X-Z-NHMe compounds corresponds to the CO@X and HN@Ala atoms in the Ala-containing peptides in such a way that the X-Ala amide group becomes slightly more polarized in the *trans* state. These small variations in atomic charges have a significant effect on the self-atomic energies and the pairwise exchange-correlation energies and, consequently, the X-Ala peptide linkages show the largest changes in the atomic distribution of the IQA magnitudes, while the Pro-containing counterparts are less affected. Notwithstanding, the terminal amide groups in Ac-X-Z-NHMe also experience changes in the Δ*E*_T,xc_ values (*e.g*., the average atomic Δ*E*_T,xc_ is around 1 kcal/mol in absolute value).

The importance of the noncovalent bonding is clearly seen in the atomic distribution of the electrostatic component (Δ*E*_elec_), which presents the higher mean atomic values (from ∼1.5 to ∼3.5 kcal/mol in absolute value) and is concentrated on the C, N, H and O atoms of all the polar amide groups, comprising the rotating X–Z bond and the other groups participating in direct H-bond or through-space electrostatic interactions. Interestingly, the electronic fingerprint of the C­(π)···HC interactions can be detected in the positive Δ*E*_T,xc_ values assigned to the aromatic C atoms in Ac-Tyr-Z-NHMe. We also see in Figure 3 that the dispersion energy change (Δ*E*_D3_) is evenly distributed over polar and nonpolar groups due to its nonspecific character. In contrast, the solvation energy rearrangement related with the *cis* → *trans* isomerization is preferentially located on the polar groups and the larger atomic contributions to Δ*E*_solv_ correspond generally to the N/O atoms that undergo greater changes in their electronic population.

The atomic IQA terms of the *zwitterionic* Leu-Z systems give convincing evidence about the predominant role of the charged groups, −COO^–^ and −NH_3_^+^, in determining the *cis–trans* energetics. Hence, the quantum Δ*E*_T,xc_, electrostatic Δ*E*_elec_ and solvation Δ*E*_solv_ values for Leu-Pro are mostly concentrated on the ionic groups so that the global Δ*E* basically measures the stability of the −COO^–^···^+^H_3_N– interaction. For Leu-Ala, the rotating amide group is also significantly affected, contributing thus to the *cis*–*trans* energy.

The widespread distribution of the atomic IQA quantities points out that the relative stability of the *cis–trans* isomers is not controlled alone by the rotating amide bond. To better understand this point, we grouped a selection of atomic and pairwise IQA energies, excluding dispersion and solvation terms, into fragment-based contributions for a six-atom fragment comprising the O,C,Cα@Ac/X and Cα,(H/Cδ),N@Z atoms of the isomerizing bond and for the 8-atom fragments (CH_3_CONH– and −CONHCH_3_) associated with the terminal amide groups (*i.e*., Ac-X and Z-NHMe). Thus, we see in Table 5 that the change in the sum of the Δ*E*_T,xc_ and Δ*E*_elec_ components for the rotating amide bond increases a few kcal/mol ongoing from the *cis* to the *trans* state, that is, its intrinsic stability is lower in the *trans* configuration, what may be compatible with its role as electron donor in the *n* → π* interactions with the neighboring amide groups. However, such destabilization of the X-Z (or Ac-Z) linkage is accompanied by relevant changes in the fragment energies of the terminal backbone groups, which tend to be more stable in the *trans* configuration (see Table 5). In addition, the total IQA interaction energy (due to covalent and noncovalent binding) among these fragments tends also to be more favorable in the *trans* disposition. Overall, this fragmentation analysis stresses that the changes in the kinetic-exchange Δ*E*_T,xc_ and electrostatic Δ*E*_elec_ energies are dominated by the electronic rearrangement within the Ac-X, X-Z (or Ac-Z) and Z-NHMe amide bonds as well as by their mutual interactions that are reinforced in the *trans* state of the X-Z bond what, in turn, can be considered as the key factor stabilizing the full *trans* state of the model peptides.

**Table 5:** Average Values of the Fragment IQA Δ*E*_T,xc_ + Δ*E*_elec_ Energies (in kcal/mol) Associated with the CαCON­(H/Cδ)­Cα Atoms of the Peptide Bond Involved in the *cis-trans* Isomerization and the Terminal Backbone Groups (CH_3_CONH and CONHCH_3_)­^a^

	Ac-Z-NHMe		Ac-Gln-Z-NHMe		Ac-Leu-Z-NHMe		Ac-Tyr-Z-NHMe		Leu-Z	
	Z = Pro	Z = Ala	Z = Pro	Z = Ala	Z = Pro	Z = Ala	Z = Pro	Z = Ala	Z = Pro	Z = Ala
	rotating amide fragment									
Δ*E*_T,xc_ + Δ*E*_elec_	2.5	2.0	3.1	6.8	4.4	8.3	2.3	5.8	0.0	3.4
	rotating amide fragment									
Δ*E*_T,xc_ + Δ*E*_elec_	–0.1	–0.2	–5.8	–4.0	–6.7	–4.0	–0.3	–0.8		
	interaction among fragments									
Δ*E*_int,elec+xc_	–3.8	–1.6	0.6	–2.6	–2.4	–3.2	–2.1	–1.8		

a The full interaction energy among the fragments is also indicated.

Finally, we also segregated the physical energetic components (Δ*E*_T,xc_, Δ*E*_elec_ and Δ*E*_D3_) into atomic contributions and pairwise (1–2, 1–3, ···) interactions (Table S6) to discriminate between *short* (atomic, 1–2 and 1–3) and *medium* (1–4 and beyond) effects. For example, the stabilization of the *cis* isomer achieved by dispersion interactions is readily ascribed to the *medium* range (>1–4) interactions. In contrast, the *trans* stabilization measured by the sum of Δ*E*_T,xc_ and Δ*E*_elec_ is in general due to *short* range effects whereas the *medium* range contributions are unfavorable, reflecting probably the loss of intramolecular contacts in the *trans* form. However, an exception arises in the *trans* isomer of Ac-Tyr-Pro-NHMe, which gives nearly the same number of BCP contacts as the *cis* form and turns out to be stabilized moderately by *medium* range contacts instead of the *short*-range contributions.

## Discussion

The MD simulations and QM calculations reported in this work characterize in detail the structural and energetic properties involved in the *cis–trans* equilibria in aqueous solution of the selected dipeptide molecules: Ac-Z-NHMe, Ac-X-Z-NHMe, and Leu-Z with X = Gln, Leu, Tyr and Z = Pro, Ala. Using classical US-PMF calculations, we find that the Ala → Pro substitution systematically decreases the Δ*G*_*cis→trans*_ value by ∼0.4–1.1 kcal/mol in absolute value, which can be considered as a quantitative measure of the Pro *cis* effect. The same trend is observed in the average QM energies at the HF-D3/cc-pVTZ SMD level computed on the solute coordinates extracted from a set of QM/MM-relaxed MD frames, which result in Δ*E*_*cis*→*trans*_ values close to the PMF Δ*G* data. The largest discrepancy between PMF and QM results is found in Ac-Tyr-Pro-NHMe for which the QM calculations predict nearly isoenergetic *cis*–*trans* isomers whereas the *trans* form would be 2.0 kcal/mol more stable according to the PMF calculations. It turns out that the Tyr side chain gives specific C­(π)···HC contacts in the *cis* isomers and although a similar Amber force field (ff14SB-TIP3P) has been found to describe quantitatively this interaction,^68^ a minor imbalance in the description of the C­(π)···HC interactions using MM or HF-D3 methods could be behind this discrepancy. Nevertheless, the rest of comparable US-PMF and QM energies suggest that *cis*–*trans* isomerization may be reasonably described using MM force fields basing on electrostatic and van der Waals parameters as well as on torsional potentials (typically derived from QM energy profiles to capture implicitly various electronic and steric effects).

Before discussing the nature of the Pro *cis* effect, it may be convenient to compare our computational results with relevant experimental and/or molecular modeling information. For the Ac-Pro-NHMe and Leu-Pro systems, the theoretical Δ*G*/Δ*E* energies can be directly compared with experimental data (see Table S7). On the one hand, from intensities of selected NMR chemical shifts in D_2_O, *trans-*Ac-Pro-NHMe has been reported to be only 0.57 kcal/mol more stable at 25 °C than the *cis* isomer.^69^ Both the US-PMF and QM calculations reproduce qualitatively this preference, albeit they overestimate the Δ*G* value by ∼0.8 and ∼0.2 kcal/mol, respectively, and have moderate statistical uncertainties (∼0.1–0.2 kcal/mol). Former calculations on Ac-Pro-NHMe using the Amber03 FF and metadynamics protocols have rendered a similar value (Δ*G*_*cis→trans*_ = −1.0 ± 0.3 kcal/mol).^70^ On the other one, the calculated Δ*G*_*cis→trans*_^US^ and Δ*E*_*cis*→*trans*_^HF‑D3^ values for the *zwitterionic* Leu-Pro, 0.5 ± 0.1 and 0.9 (0.2) kcal/mol, respectively, admit direct comparison with the −0.02 kcal/mol value obtained from NMR measurements at pH 6.5 at which the *zwitterionic* form is dominant (we note in passing that alkaline pH stabilizes the *cis* form of the X-Pro dipeptides; see Table S7).^20^

In the Supporting Information (Table S7), we summarize other computational results as well as additional experimental *cis*-*trans* energies for closely related molecules (Ac-NHMe, Ac-Ala-X-Pro-Ala-Lys-NH_2_, *etc.*). Thus, it turns out that the average QM energies in Table 1 for Ac-Z-NHMe are nearly identical to previous DFT calculations,^15^ while approximated MM calculations on the Ac-X-Pro-NHMe systems^14^ predict *trans* preferences that are not far (<0.7 kcal/mol) from the US-PMF ones. Concerning the experimental studies on other oligopeptide systems with X-Pro linkages (X = Gly, Gln, Tyr, Phe),^11^ their Δ*G*_*cis→trans*_ values are also small in absolute value (<1.2 kcal/mol), showing thus the propensity of these systems to populate both the *trans* and *cis* isomeric states.

Considering that (i) the differences between computational and experimental values are under 1 kcal/mol, which is considered as the threshold of chemical accuracy in computational chemistry; (ii) the accuracy of the US-PMF calculations is also comparable to that of relative binding free energy calculations (∼1 kcal/mol) using the same ff19SB force field.;^71^ and (iii) the *cis–trans* preferences of the examined model systems are in consonance with other experimental and theoretical data, we expect that the comparative IQA analysis of the conformationally averaged QM energies of the Pro- and Ala-containing dipeptides may yield new insight into the origin of the Pro *cis* effect. Since this effect is due to a small energy difference (<1 or 2 kcal/mol), we focused on the analysis of the average magnitudes derived from independent IQA calculations over at least 100 structures.

We evaluated first the IQA-based descriptors for steric hindrance to assess the relative weight of steric repulsions involving the −CαH– and −CδH_2_– groups, which are commonly assumed as the key factor explaining the Pro *cis* effect. Although the sign of these fragment contributions to the *E*_ST_ descriptor are in consonance with the expected release and accumulation of steric strain upon *cis–trans* isomerization, they do not provide a clear rationale for the Pro *cis* effect because other atomic positions present comparable *E*_ST_ values that, in turn, can be affected by diverse electronic/electrostatic effects. Likewise, we confirmed the presence and stabilizing nature of the C=O···C=O interactions in the *trans* isomers by examining the exchange interaction energies (Δ*E*_int,xc_) involving the carbonyl at the X-Z linkage and the subsequent backbone carbonyl group. However, this effect, which admits an orbital description in terms of *n* → π* hyperconjugation, is accompanied by many other deformation, electrostatic and solvation changes as pointed out by the IQA decomposition. Moreover, its variability with the peptide sequence does not explain either the Pro *cis* effect.

To adopt a broader and simpler view of the energetic analysis of isomerization equilibria, we propose to group the atomic and diatomic IQA quantities into four contributions: quantum (Δ*E*_T,xc_,which embodies steric repulsion, hyperconjugation, *etc.*), electrostatic (Δ*E*_elec_), dispersion (Δ*E*_D3_) and solvation (Δ*E*_solv_). It turns out that the pattern and magnitude of the Δ*E*_T,xc_ and Δ*E*_elec_ changes depend on the peptide sequence, what is also seen in their widespread atomic distribution over the isomerizing amide group and the rest of functional groups involved in the formation/rupture of diverse intramolecular contacts. Nonetheless, the sum of Δ*E*_T,xc_ and Δ*E*_elec_ favors systematically the *trans* isomer of the Ac-Z-NHMe and Ac-X-Z-NHMe peptides by a few kcal/mol and their fragmentation suggests that the *trans* arrangement maximizes the stabilizing interactions among the backbone amide groups. Excepting the Ac-Z-NHMe system, the *trans* stabilization due to the electronic and electrostatic effects captured by the Δ*E*_T,xc_ and Δ*E*_elec_ IQA terms turns out to be more accentuated (or nearly equal) in the Pro-containing peptides than in the Ala-containing ones.

Concerning the role played by the attractive dispersion interactions, the D3 pairwise potentials indicate that the pyrrolidine ring of the Pro residue reduces the loss of dispersion energy ongoing from the slightly more compact *cis* form to the *trans* one, what constitutes a small but systematic effect favoring the *trans* isomers of the Pro-containing peptides with reference to the Ala-systems.

The influence of solvation energy was taken into account by means of the SMD continuum model that allows us to partition the solute–solvent interaction energy into effective atomic contributions using the IQA approach. Comparison of the *cis* → *trans* changes in dipole moments (Δμ) and solute–solvent interactions (Δ*E*_solv_) for the Ac-Z-NHMe and Ac-X-Z-NHMe systems (Z = Pro, Ala) readily shows a clear relationship between solvation and the Pro *cis* effect. Thus, the –CδH_2_ group attached to the N atom of Pro (instead of the H atom of Ala) influences the polarity of the X-Pro peptide bond in such a way that the *cis* form of the Ac-Pro-NHMe and Ac-X-Pro-NHMe peptides is more polar (*i.e*., μ*_cis_* > μ*_trans_* by nearly 2 D units) whereas the opposite behavior applies to the Ac-X-Ala-NHMe systems (*i.e*., μ*_cis_* < μ*_trans_* by ∼0.1–1.0 D). These differences in the polarity of the neutral Pro and Ala-systems are correlated with the *cis* → *trans* Δ*E*_solv_ energies that result in the preferential stabilization of the *cis* isomer of the Pro-containing dipeptides by at least ∼1 kcal/mol.

At this point, one may wonder to what extent the solvent effects underlined by the energy decomposition analysis are supported by previous studies. To answer this question, we focus on results reported for identical or similar oligopeptides given that solvent effects largely depend on the solute size and its hydrophobic/hydrophilic properties. For example, ^1^H NMR experiments^18^ have shown that the fraction of *cis*-Ac-Pro-NMe_2_ is reduced from 20% in water to 10% in chloroform. In the same work, DFT methods coupled with a continuum solvation model assign a larger polarity to *cis*-Ac-Pro-NMe_2_ than to the *trans* isomer. The *cis:trans* ratio of Ac-Pro-NHMe has also been determined at various *T* and solvent conditions using ^1^H NMR.^72^ These experiments indicate that the *cis* isomer tends to be more abundant in the polar solvents than in the nonpolar ones (*e.g*., 27% in water at 57 °C *vs*, 6% in CDCl_3_ at 36 °C). This trend has been reproduced by QM calculations^73^ predicting a higher population of the *cis* isomer in the more polar solvents. Similarly, ^13^C NMR measurements performed for the Ac-Gly-Pro-OMe peptide at 25 °C have detected a small decrease of *cis* population in toluene (7%) with respect to that in water (9%).^74^ Altogether these results point toward a more polar *cis* X-Pro isomer in the case of small and Ac/NHme capped systems so that polar solvents can stabilize the *cis* configuration as suggested by our results.

Besides the Ac-Z-NHMe and Ac-X-Z-NHMe peptides bearing neutral caps, we considered one *zwitterionic* system (Leu-Z, Z = Ala or Pro) to analyze the influence of the vicinal charged groups on the *cis*–*trans* equilibrium. Our results indicate that the Ala → Pro substitution reinforces the strength of the carboxylate···ammonium salt bridge that forms exclusively in the *cis* configuration and that the *cis* isomer may become more populated in aqueous solution according to the PMF-US and QM isomeric energies. Actually, ^13^C NMR spectra reveal that the *cis*–*trans* isomers of X-Pro *zwitterions* (X = Ala, Leu) have nearly equal population in aqueous solution (see Table S7).^20^ Solvent effects in these *zwitterionic* X-Pro systems are opposite to those observed for the capped dipeptides because the population of the *cis* isomer increases ongoing from water to methanol and dimethyl sulfoxide.^20^ This is in line with our analysis showing that solvation energy is the main contribution to the stability of the *trans* Leu-Pro. The importance of the interactions between the *N*- and *C*-terminal groups has been also emphasized by recent multidimensional NMR experiments^75^ showing that charge neutralization of short peptides (Phe-Pro-Ala, Met-Pro-Ser, and Ala-Pro-Gln) by *N*-terminal acetylation and *C*-terminal amidation decreases the stability of their *cis* isomers with respect to those of the *zwitterionic* forms.

Finally, we can summarize our findings as follows: (i) a combination of electrostatic and hyperconjugative interactions promote the *trans* state in the capped dipeptides, but these *trans* effects are modulated by the amino acid sequence and do not allow a simple interpretation of the propensity of native Pro residues to adopt the *cis* isomeric state; (ii) a more systematic role is played by solvation effects to control the *cis*–*trans* energy gap of the Pro-containing dipeptides. However, this rationale of the Pro *cis* effect in short oligopeptides cannot be readily extended to more complex biomolecules such as IDPs. Thus, NMR analysis has demonstrated that the population of *cis*-Pro in -X-X-Pro-X segments within unfolded proteins is below estimates obtained for small peptides bearing the same amino acid sequence,^75^ indicating thus that environment effects disfavor the *cis* state. A complex interplay of effects is also observed in the case of polyproline peptides, (Pro)_*n*_, which fold in different helical secondary structures, ranging from the all-*trans* PPII helix to the all-*cis* PPI helix. Conversion among these forms occurs through *cis–trans* isomeric changes that are induced by temperature, chain-length, solvent, concentration and the presence of other amino acids in the peptide chain.^76–78^ Interestingly, *trans* Pro-Pro bonds in polyproline are favored in polar solvents thanks to entropy effects whereas *cis* Pro-Pro linkages are enthalpically favored in nonpolar solvents,^77^ what contrasts with the preferences exhibited by the capped dipeptides. Therefore, it turns out that, to better understand *cis–trans* isomerization of Pro residues in polyproline or IDPs, a detailed analysis of intramolecular and solute–solvent interactions including molecular entropy estimations would be required. Eventually, this challenge may be addressed by computational strategies combining extensive conformational sampling, free energy and molecular entropy calculations as well as QM calculations complemented with energy decomposition analysis.

## Supplementary Material



## Data Availability

Cartesian coordinates and IQA results in csv format are available at the Mendeley data repository (10.17632/z5tf8jp3kg.1).

## Notes

US-PMF free energy profiles; conformational entropy plots. Ionization potentials employed to estimate steric energies; HF-D3 and DLPNO–CCSD­(T) *cis–trans* energies in the gas-phase for selected systems; ring puckering conformation data; Molecular surface area; Ramachandran plots; BCP properties and NCI plots; short and medium range IQA contributions to the *cis–trans* isomerization energy; summary of experimental and previous theoretical results (PDF)
