# Supplementary material for: Assessment of Energy Effects Determining cis-trans Proline Isomerization in Dipeptides
Source: ACS Phys Chem Au. 2025 Dec 4;6(1):38–56. doi: 10.1021/acsphyschemau.5c00072 (PMC12856670; doi:10.1021/acsphyschemau.5c00072)
Supplement: Supplementary file 1 [file pg5c00072_si_001.pdf]

Assessment of energy effects determining *cis*-*trans* Proline isomerization in Dipeptides

Natalia Díaz,[a] *Roberto López*[b]*, Ángel Martín-Pendás* [a] *and Dimas Suárez* [a]*

(a) Departamento de Química Física y Analítica. Universidad de Oviedo. Facultad de Química.

Avda. Julián Clavería 8. 33006 Oviedo (Asturias) Spain.

(b) Departamento de Química y Física Aplicadas. Universidad de León. Facultad de Biología.

Campus de Vegazana s/n. 24071 León (Castilla y León) Spain

*E-mail: dimas@uniovi.es

Supporting Information

Figure S1. US-PMF free energy profiles (in kcal/mol) for the *cis→trans* isomerization of the model systems studied in this work.

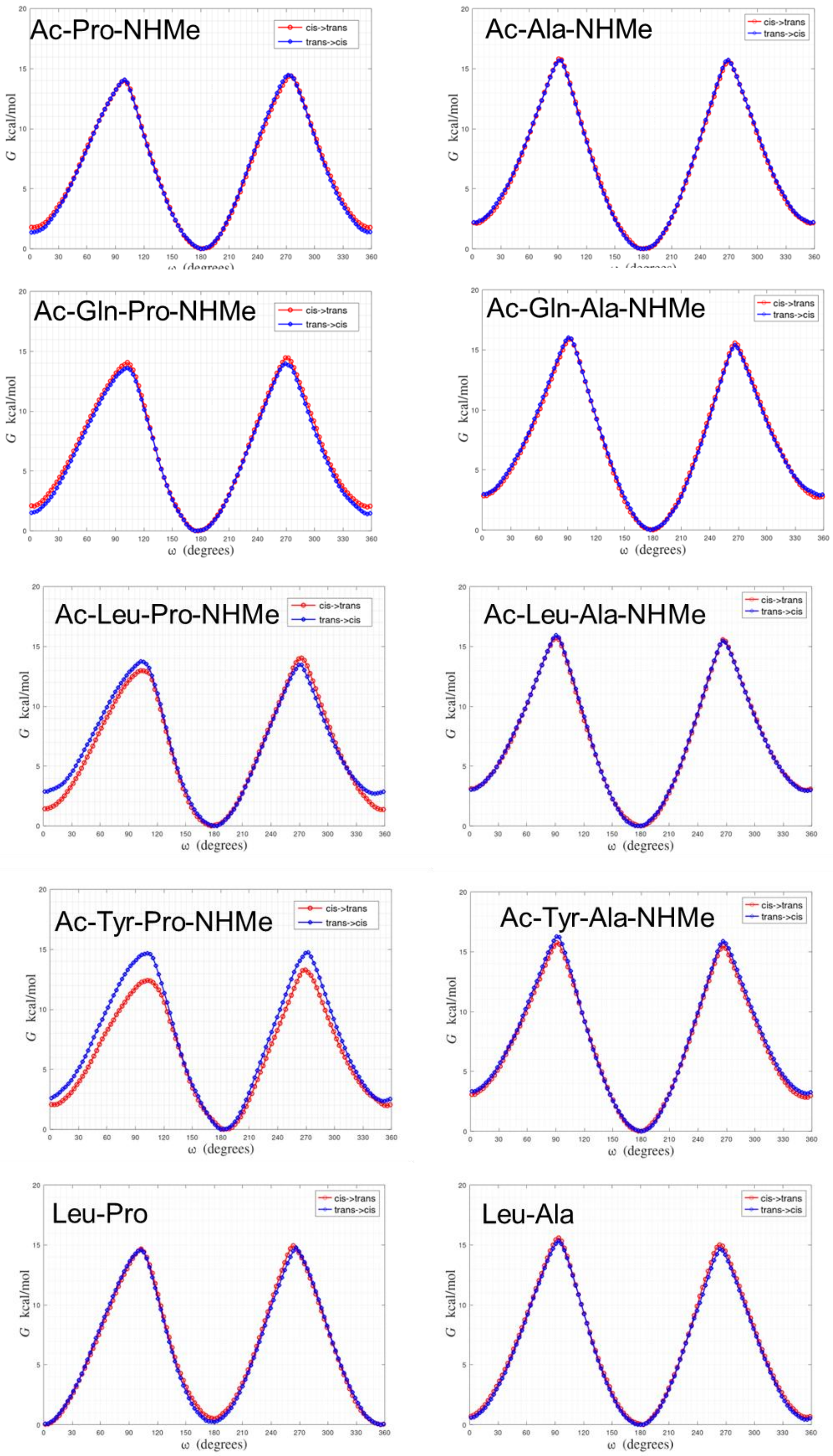

Figure S2. Convergence plots of conformational entropy ($-TS_{conform}$ in kcal/mol) for the different model systems as calculated with the CENCALC_QUICKSORT program. The average value and standard deviation of the $-TS_{conform}$ values from 375 to 500 ns are also indicated.

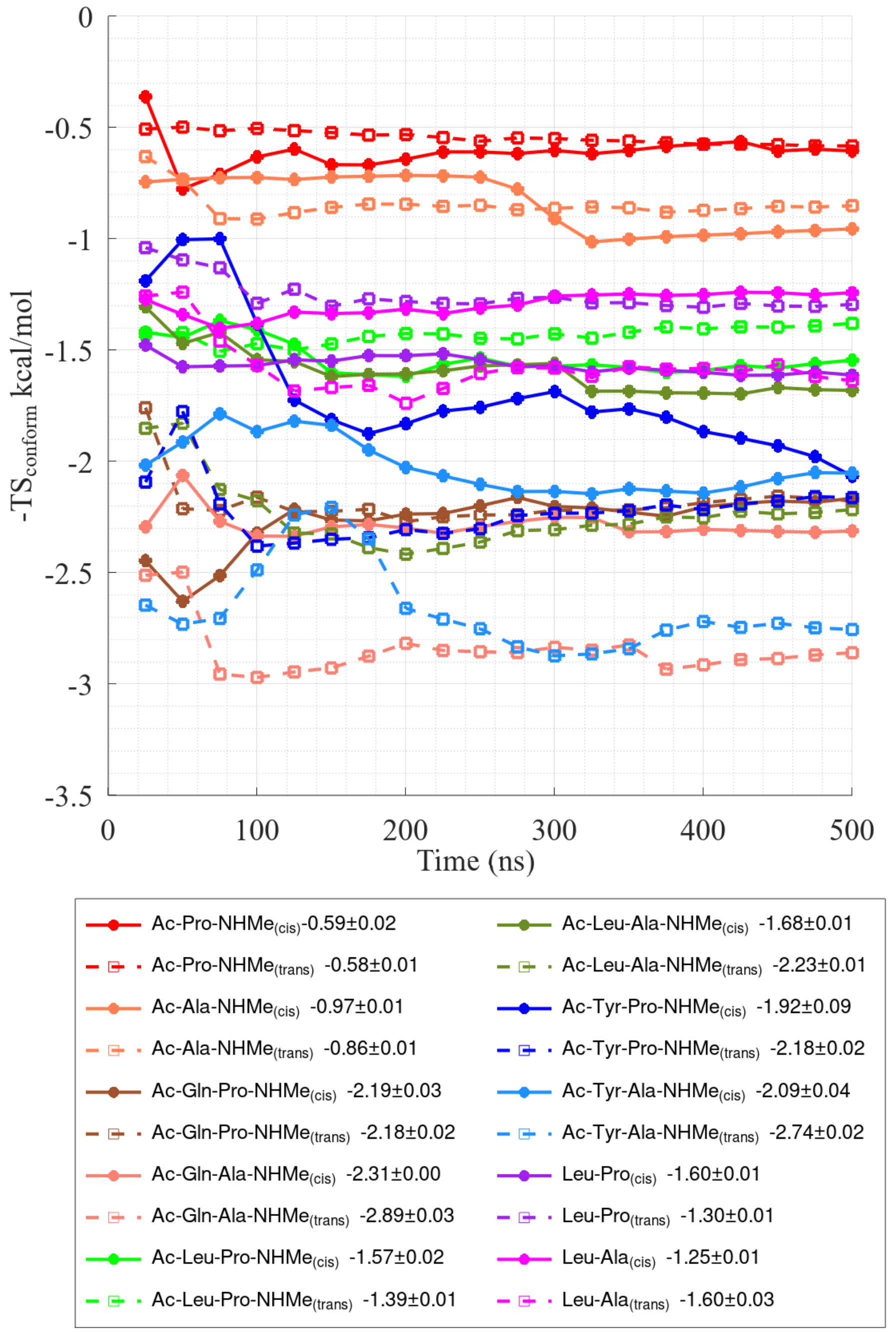

Table S1. Ionization potentials (in kcal/mol) used to estimate the IQA steric energy values.

Element X	$X^{-2} \rightarrow X^{-1}$	$X^{-1} \rightarrow X^{0}$	$X^{0} \rightarrow X^{+1}$	$X^{+1} \rightarrow X^{+2}$	$X^{+2} \rightarrow X^{+3}$
H [(a)]		17.4	313.58		
C [(a)]		29.166	259.7	562.42	1104.4
N [(b)]	-161				
O [(b)]	-178				

(a) Experimental values: Handbook of Chemistry and Physics. W.M. Haynes, Ed. 93rd edition. CRC Press 2012.

(b) Calculated values: Pearson, R.G. *Inorg. Chem.* **1991**, *30*, 2856-2858.

Table S2 *cis-trans* energies in the **gas-phase** for the Ac-Z-NHMe and Ac-Gln-Z-NHMe peptides obtained from average HF-D3/cc-pVTZ and DLPNO-CCSD(T)/cc-pVTZ energies. The $\Delta E_{cis \rightarrow trans}$ values are in kcal/mol (standard errors in parentheses).

System	$\Delta E_{cis \rightarrow trans}^{HF-D3}$	$\Delta E_{cis \rightarrow trans}^{DLPNO-CCSD(T)}$
Ac-Pro-NHMe	-1.30 (0.18)	-1.13(0.16)
Ac-Ala-NHMe	-2.76(0.23)	-2.87(0.21)
Ac-Gln-Pro-NHMe	0.07(0.56)	0.24(0.48)
Ac-Gln-Ala-NHMe	-1.66(0.60)	-1.75(0.51)

Table S3 Percentage of abundance of the Cγ(*endo*) /Cγ(*exo*) ring puckering conformation of Pro residues determined by the CPPTRAJ program using the Cremer convention (phase angle θ<0 ↔Cγ(*endo*) and θ>0 ↔Cγ(*exo*)).

			Ac-X-Pro-NHMe							
**Puckering**	**Ac-Pro-NHMe**		**X=Gln**		**X=Leu**		**X=Tyr**		**Leu-Pro**	
	cis	*trans*	*cis*	*trans*	*cis*	*trans*	*cis*	*trans*	*cis*	*trans*
Cγ(*endo*)	66%	59%	71%	58%	69%	61%	66%	62%	56%	46%
Cγ(*exo*)	34%	41%	29%	42%	31%	39%	34%	38%	44%	54%

Table S4. Average value of the solvent excluded surface (SES in Å^2) and radius of gyration (R_{gyr} in Å) of the dipeptide molecules obtained from the MD simulations.

System	SES *cis/trans*	R_{gyr} *cis/trans*
Ac-Pro-NHMe	213.4 /216.2	2.82±0.04 / 2.94±0.06
Ac-Ala-NHMe	192.0 / 197.0	2.71±0.09 / 2.85±0.08
Ac-Gln-Pro-NHMe	319.2 / 329.4	3.62±0.11 / 3.86±0.11
Ac-Gln-Ala-NHMe	301.4 / 314.0	3.57±0.15 / 3.85±0.15
Ac-Leu-Pro-NHMe	328.3 / 339.4	3.62±0.05 / 3.87±0.08
Ac-Leu-Ala-NHMe	309.8 / 323.5	3.56±0.11 / 3.85±0.12
Ac-Tyr-Pro-NHMe	319.1/ 323.4	3.76±0.09 / 3.91±0.17
Ac-Tyr-Ala-NHMe	305.0 / 310.6	3.79±0.13 / 3.95±0.20
Leu-Pro	263.0 / 266.8	3.23±0.07 / 3.29±0.20
Leu-Ala	242.6 / 252.0	3.05±0.07 / 3.28±0.11

Figure S3. Ramachandran plots of the φ and ψ backbone torsions derived from the MD simulations. Criteria for defining secondary structure: PPII helix−104° ≤ φ ≤ −46° and 116° ≤ ψ ≤ 174°; β-structures −180° ≤ φ <−104° and 104° ≤ ψ ≤ 180°: right-handed helices −90° ≤ φ ≤ −30° and −90° ≤ ψ ≤ 0°; the remaining values would be considered random coil.

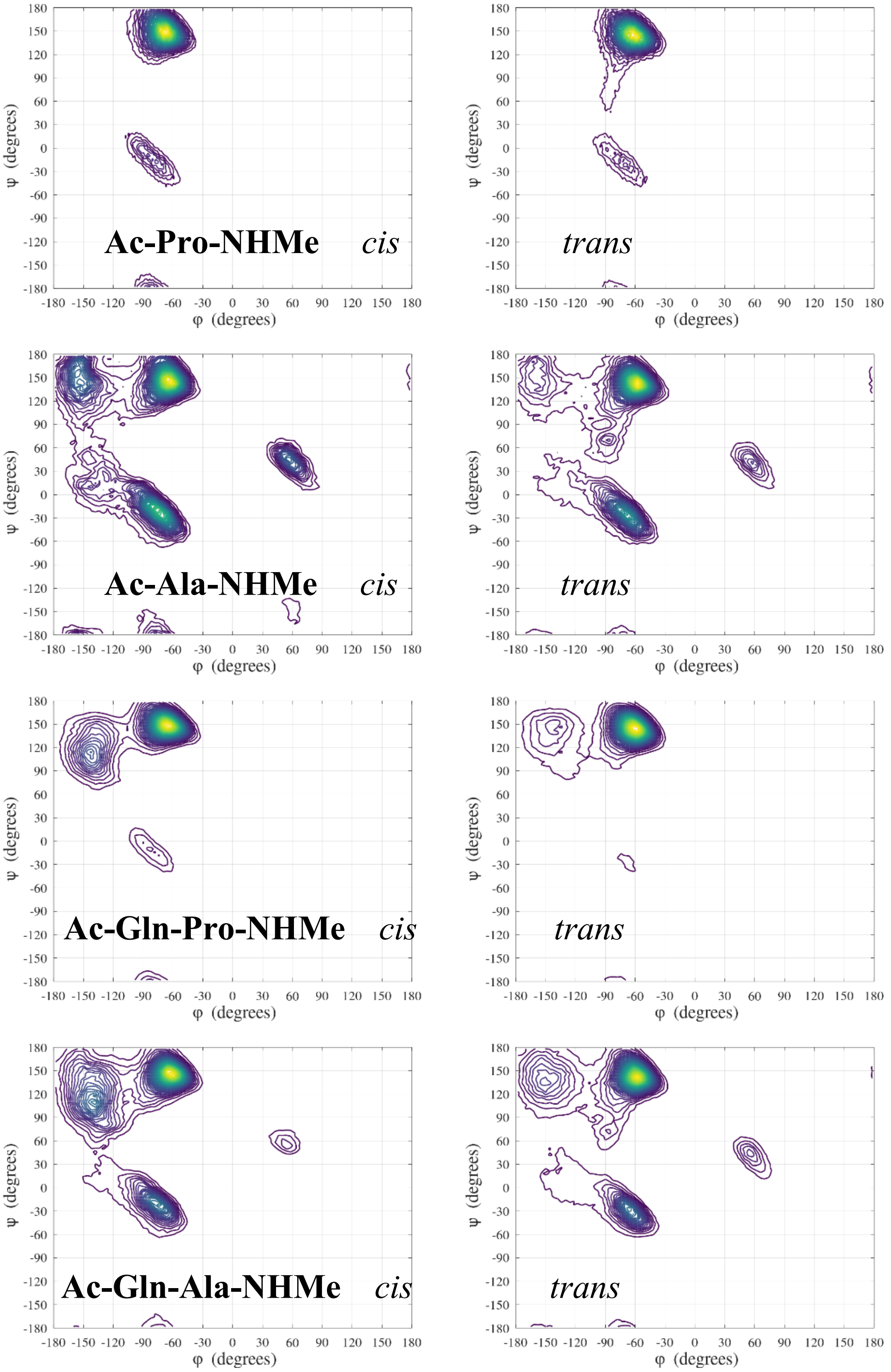

Figure S3 (cont.)

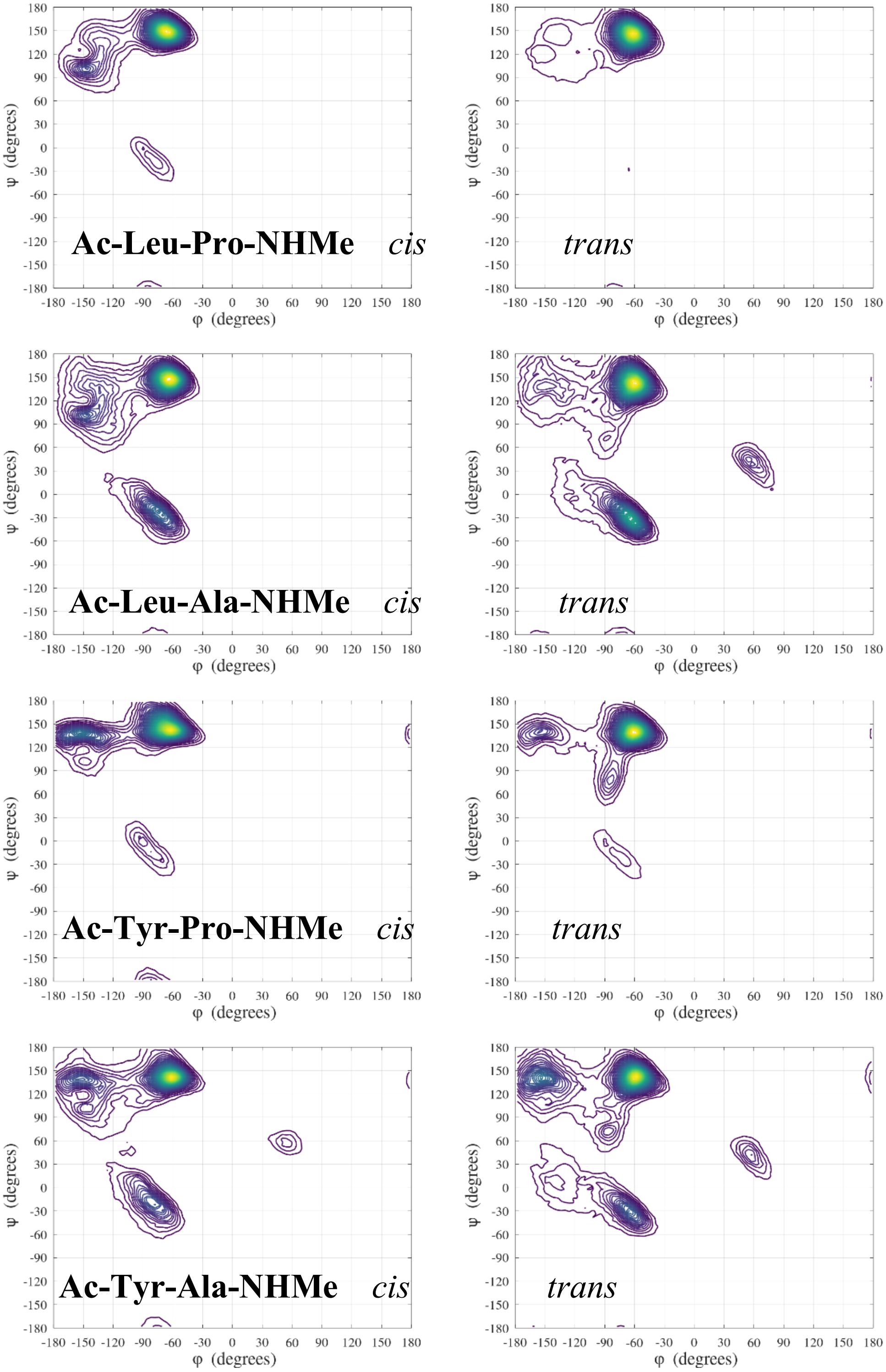

Figure S3 (cont.)

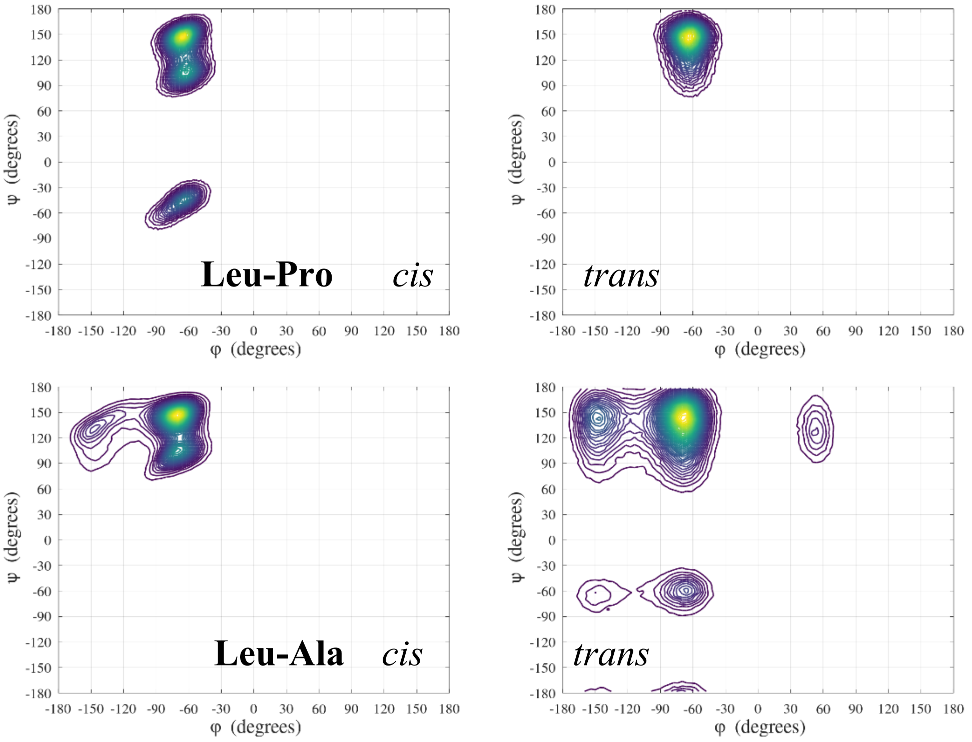

Table S5. Average Bond-Critical-Point (BCP) properties of ρ(r) for noncovalent contacts in the QM/MM optimized structures. BCP properties were obtained from HF/cc-pVTZ SMD charge densities. r_c, $\rho(r_c)$, $\nabla^2\rho(r_c)$ and ε stand for the BCP, the electronic density, the Laplacian of the electronic density and ellipticity at the BCP, respectively. r_1 and r_2 are the bond path lengths from the critical point to A and B in the A-B bond. $H(r_c)$ is the local energy density. Only BCPs with abundance above 5% are shown.

A		B		%	$\rho(r_c)$	$\nabla^2\rho$	ε	r_1	r_2	$H(r_c)$
					**Ac-Pro-NHMe *cis***					
H1	ACE	O	PRO	13	0.0087	0.0354	1.104	1.232	1.612	-0.0056
H2	ACE	O	PRO	8	0.0094	0.0384	1.213	1.213	1.711	-0.0062
H3	ACE	N	NME	7	0.0084	0.0325	1.250	1.143	1.706	-0.0049
HG2	PRO	O	PRO	35	0.0092	0.0380	0.840	1.213	1.502	-0.0061
					**Ac-Pro-NHMe *trans***					
HG2	PRO	O	PRO	31	0.0091	0.0372	0.710	1.218	1.505	-0.0059
					**Ac-Ala-NHMe *cis***					
H1	ACE	O	ALA	6	0.0094	0.0379	1.410	1.194	1.734	-0.0061
H1	ACE	N	NME	6	0.0079	0.0304	1.004	1.154	1.647	-0.0044
					**Ac-Ala-NHMe *trans***					
O	ACE	H	NME	5	0.0179	0.0765	0.093	1.336	0.819	-0.0135
					**Ac-Gln-Pro-NHMe *cis***					
O	ACE	HB2	GLN	5	0.0110	0.0480	1.195	1.456	1.171	-0.0077
O	ACE	HG2	GLN	9	0.0086	0.0355	0.610	1.523	1.194	-0.0056
O	ACE	NE2	GLN	6	0.0058	0.0228	1.790	1.632	1.776	-0.0034
O	ACE	O	PRO	22	0.0045	0.0170	0.952	1.669	1.716	-0.0028
O	ACE	N	NME	30	0.0056	0.0216	1.200	1.650	1.703	-0.0032
O	ACE	H1	NME	10	0.0044	0.0170	0.321	1.700	1.292	-0.0026
O	ACE	H2	NME	12	0.0043	0.0172	0.545	1.703	1.307	-0.0026
O	ACE	H3	NME	13	0.0041	0.0158	0.340	1.688	1.322	-0.0024
N	GLN	O	PRO	21	0.0072	0.0280	0.769	1.628	1.703	-0.0042
N	GLN	N	NME	7	0.0067	0.0253	0.777	1.643	1.715	-0.0036
HA	GLN	OE1	GLN	17	0.0117	0.0511	0.604	1.092	1.442	-0.0081
HA	GLN	CA	PRO	11	0.0148	0.0636	5.562	1.134	1.666	-0.0116
HA	GLN	HA	PRO	22	0.0153	0.0617	1.110	1.091	1.237	-0.0110
HA	GLN	C	PRO	6	0.0143	0.0571	1.678	1.015	1.444	-0.0107
HB2	GLN	HA	PRO	9	0.0105	0.0398	0.703	1.126	1.064	-0.0064
HB3	GLN	HA	PRO	18	0.0094	0.0374	0.671	1.212	1.103	-0.0059
HG2	GLN	O	GLN	5	0.0120	0.0512	0.679	1.148	1.439	-0.0082
HG3	GLN	O	GLN	9	0.0108	0.0465	1.917	1.186	1.477	-0.0074
OE1	GLN	HA	PRO	26	0.0099	0.0399	0.282	1.492	1.019	-0.0065
OE1	GLN	H	NME	12	0.0132	0.0566	0.140	1.414	0.950	-0.0101
NE2	GLN	HG3	PRO	7	0.0041	0.0144	0.333	1.822	1.318	-0.0021
NE2	GLN	HA	PRO	7	0.0069	0.0257	0.687	1.714	1.135	-0.0040
HG2	PRO	O	PRO	36	0.0098	0.0403	0.588	1.192	1.486	-0.0064

Ac-Gln-Pro-NHMe *trans*

A		B		%	ρ(*r*c)	∇2ρ	ε	*r*1	*r*2	*H*(*r*c)
O	ACE	HD3	PRO	24	0.0075	0.0294	0.393	1.551	1.147	-0.0047
H	GLN	OE1	GLN	6	0.0216	0.0879	0.301	0.831	1.310	-0.0178
HA	GLN	OE1	GLN	23	0.0107	0.0459	1.001	1.150	1.465	-0.0073
HA	GLN	CD	PRO	10	0.0150	0.0653	2.481	1.108	1.661	-0.0117
HA	GLN	HD2	PRO	5	0.0143	0.0594	2.134	1.141	1.294	-0.0104
HA	GLN	HD3	PRO	35	0.0149	0.0601	1.020	1.072	1.230	-0.0105
HB2	GLN	HD2	PRO	7	0.0094	0.0360	0.487	1.110	1.217	-0.0057
HB3	GLN	HD2	PRO	22	0.0095	0.0374	0.860	1.155	1.184	-0.0060
HG2	GLN	O	PRO	14	0.0067	0.0255	0.124	1.121	1.600	-0.0041
HG3	GLN	HD2	PRO	8	0.0059	0.0230	0.412	1.315	1.216	-0.0034
HG3	GLN	O	PRO	22	0.0061	0.0233	0.259	1.155	1.628	-0.0037
OE1	GLN	HD2	PRO	24	0.0080	0.0309	0.237	1.556	1.084	-0.0050
NE2	GLN	HD2	PRO	10	0.0064	0.0235	1.156	1.749	1.161	-0.0036
NE2	GLN	O	PRO	5	0.0042	0.0162	0.900	1.875	1.695	-0.0024
HG2	PRO	O	PRO	30	0.0090	0.0369	0.722	1.210	1.506	-0.0059

Ac-Gln-Ala-NHMe *cis*

O	ACE	HB2	GLN	8	0.0115	0.0489	0.750	1.443	1.148	-0.0079
O	ACE	HG2	GLN	6	0.0091	0.0379	0.647	1.523	1.215	-0.0060
O	ACE	HA	ALA	8	0.0102	0.0411	0.218	1.482	1.040	-0.0067
O	ACE	O	ALA	15	0.0046	0.0176	0.906	1.670	1.726	-0.0028
O	ACE	N	NME	25	0.0060	0.0235	0.977	1.635	1.710	-0.0035
O	ACE	H	NME	9	0.0111	0.0477	0.287	1.453	1.056	-0.0078
O	ACE	H1	NME	8	0.0041	0.0165	0.468	1.687	1.328	-0.0025
N	GLN	HA	ALA	7	0.0145	0.0604	0.278	1.419	1.066	-0.0102
N	GLN	O	ALA	7	0.0065	0.0254	1.413	1.670	1.687	-0.0038
N	GLN	N	NME	12	0.0064	0.0235	0.863	1.662	1.688	-0.0034
HA	GLN	OE1	GLN	24	0.0113	0.0487	0.590	1.100	1.447	-0.0077
HA	GLN	CA	ALA	8	0.0149	0.0653	2.623	1.147	1.645	-0.0119
HA	GLN	HA	ALA	48	0.0149	0.0605	0.959	1.129	1.177	-0.0106
HB2	GLN	HA	ALA	8	0.0112	0.0425	0.413	1.117	1.054	-0.0071
HB3	GLN	HA	ALA	13	0.0096	0.0375	0.515	1.186	1.079	-0.0060
OE1	GLN	HA	ALA	22	0.0098	0.0393	0.247	1.506	1.014	-0.0064

Ac-Gln-Ala-NHMe *trans*

O	ACE	O	ALA	6	0.0052	0.0200	0.298	1.652	1.661	-0.0032
O	ACE	H	NME	13	0.0135	0.0566	0.113	1.411	0.905	-0.0105
HA	GLN	OE1	GLN	17	0.0122	0.0526	0.631	1.110	1.429	-0.0085
HG2	GLN	O	GLN	6	0.0109	0.0464	1.014	1.162	1.478	-0.0073
HG2	GLN	O	ALA	7	0.0051	0.0188	0.303	1.198	1.669	-0.0030
HG3	GLN	N	ALA	6	0.0102	0.0410	0.950	1.210	1.563	-0.0063
HG3	GLN	O	ALA	10	0.0077	0.0298	0.187	1.078	1.555	-0.0048

Ac-Leu-Pro-NHMe *cis*

A		B		%	$\rho(r_c)$	$\nabla^2\rho$	ε	r_1	r_2	$H(r_c)$
O	ACE	HB2	LEU	6	0.0109	0.0469	0.843	1.457	1.150	-0.0076
O	ACE	HG	LEU	10	0.0084	0.0336	0.887	1.540	1.134	-0.0054
O	ACE	HD23	LEU	8	0.0083	0.0316	0.291	1.523	1.100	-0.0052
O	ACE	O	PRO	33	0.0047	0.0174	0.470	1.666	1.689	-0.0029
O	ACE	N	NME	31	0.0059	0.0230	0.772	1.628	1.707	-0.0035
O	ACE	H1	NME	11	0.0042	0.0167	0.710	1.700	1.338	-0.0025
O	ACE	H2	NME	10	0.0044	0.0172	0.516	1.691	1.322	-0.0026
O	ACE	H3	NME	12	0.0046	0.0183	0.468	1.677	1.302	-0.0028
N	LEU	O	PRO	25	0.0076	0.0300	0.745	1.614	1.658	-0.0046
HA	LEU	CA	PRO	7	0.0153	0.0648	3.729	1.099	1.664	-0.0119
HA	LEU	HA	PRO	16	0.0149	0.0601	1.223	1.089	1.247	-0.0106
HA	LEU	C	PRO	6	0.0121	0.0505	4.640	1.068	1.556	-0.0088
HB3	LEU	HA	PRO	40	0.0101	0.0385	0.537	1.155	1.087	-0.0062
HD11	LEU	HA	PRO	7	0.0082	0.0298	0.315	1.104	1.099	-0.0049
HD12	LEU	HA	PRO	8	0.0085	0.0304	0.244	1.089	1.078	-0.0050
HD13	LEU	HA	PRO	6	0.0076	0.0277	0.761	1.123	1.113	-0.0044
HD21	LEU	N	NME	9	0.0053	0.0190	0.323	1.229	1.753	-0.0028
HD22	LEU	N	NME	11	0.0050	0.0179	0.743	1.294	1.774	-0.0026
HD23	LEU	N	NME	9	0.0048	0.0172	0.784	1.300	1.784	-0.0025
HG2	PRO	O	PRO	43	0.0095	0.0393	0.905	1.206	1.497	-0.0063
**Ac-Leu-Pro-NHMe *trans***										
O	ACE	HD3	PRO	22	0.0080	0.0316	0.296	1.539	1.123	-0.0051
HA	LEU	CD	PRO	14	0.0150	0.0649	2.846	1.092	1.675	-0.0117
HA	LEU	HD3	PRO	35	0.0152	0.0608	0.928	1.052	1.228	-0.0107
HB3	LEU	HD2	PRO	32	0.0091	0.0358	0.634	1.168	1.187	-0.0057
HG	LEU	O	PRO	17	0.0066	0.0242	0.147	1.147	1.597	-0.0040
HD11	LEU	HD2	PRO	8	0.0081	0.0296	0.276	1.125	1.157	-0.0048
HD12	LEU	HD2	PRO	7	0.0068	0.0249	0.314	1.164	1.194	-0.0039
HD13	LEU	HD2	PRO	7	0.0074	0.0269	0.202	1.147	1.139	-0.0043
HG2	PRO	O	PRO	31	0.0092	0.0377	0.815	1.215	1.503	-0.0060
**Ac-Leu-Ala-NHMe *cis***										
O	ACE	HG	LEU	7	0.0089	0.0362	0.892	1.522	1.122	-0.0059
O	ACE	HD21	LEU	6	0.0076	0.0288	0.258	1.542	1.144	-0.0047
O	ACE	HD23	LEU	8	0.0079	0.0300	0.207	1.538	1.105	-0.0049
O	ACE	O	ALA	17	0.0051	0.0196	0.599	1.641	1.685	-0.0032
O	ACE	N	NME	31	0.0057	0.0222	0.743	1.628	1.712	-0.0034
O	ACE	H	NME	8	0.0098	0.0424	0.212	1.477	1.053	-0.0066
O	ACE	H1	NME	8	0.0040	0.0159	0.439	1.691	1.330	-0.0024
O	ACE	H2	NME	11	0.0042	0.0173	0.550	1.694	1.350	-0.0026
O	ACE	H3	NME	9	0.0050	0.0203	0.497	1.635	1.301	-0.0031
N	LEU	O	ALA	9	0.0082	0.0326	0.941	1.602	1.723	-0.0050

A		B		%	ρ(r_c)	$\nabla^2\rho$	ε	r_1	r_2	$H(r_c)$
N	LEU	N	NME	7	0.0073	0.0267	0.777	1.640	1.669	-0.0039
HA	LEU	CA	ALA	10	0.0145	0.0634	2.510	1.157	1.692	-0.0114
HA	LEU	HA	ALA	38	0.0153	0.0613	0.847	1.105	1.155	-0.0108
HA	LEU	C	ALA	6	0.0128	0.0520	2.021	1.053	1.476	-0.0094
HB3	LEU	HA	ALA	33	0.0096	0.0372	0.715	1.187	1.097	-0.0060
HB3	LEU	HB3	ALA	6	0.0077	0.0278	0.241	1.147	1.130	-0.0044
				**Ac-Leu-Ala-NHMe *trans***						
O	ACE	HB2	LEU	21	0.0123	0.0527	1.069	1.424	1.174	-0.0086
O	ACE	HD23	LEU	6	0.0075	0.0282	0.162	1.547	1.110	-0.0046
O	ACE	H	NME	10	0.0133	0.0558	0.141	1.397	0.863	-0.0099
HG	LEU	O	LEU	7	0.0108	0.0449	1.262	1.140	1.478	-0.0072
HG	LEU	O	ALA	8	0.0060	0.0217	0.264	1.156	1.606	-0.0036
HD13	LEU	O	LEU	6	0.0067	0.0252	0.162	1.195	1.581	-0.0041
				**Ac-Tyr-Pro-NHMe *cis***						
O	ACE	HB2	TYR	11	0.0115	0.0497	0.868	1.443	1.127	-0.0080
O	ACE	O	PRO	28	0.0052	0.0199	0.927	1.629	1.710	-0.0032
O	ACE	N	NME	23	0.0061	0.0243	0.820	1.598	1.714	-0.0036
O	ACE	H	NME	5	0.0164	0.0735	0.176	1.338	0.877	-0.0122
O	ACE	H1	NME	7	0.0040	0.0165	0.486	1.713	1.397	-0.0025
O	ACE	H2	NME	12	0.0040	0.0158	0.343	1.687	1.319	-0.0024
O	ACE	H3	NME	16	0.0032	0.0128	0.509	1.758	1.386	-0.0019
N	TYR	O	PRO	9	0.0076	0.0302	0.784	1.606	1.640	-0.0046
HA	TYR	CA	PRO	20	0.0153	0.0653	2.805	1.138	1.684	-0.0120
HA	TYR	HA	PRO	18	0.0155	0.0626	1.001	1.095	1.274	-0.0112
CG	TYR	HA	PRO	43	0.0101	0.0331	0.925	1.769	1.052	-0.0057
CD1	TYR	N	PRO	11	0.0090	0.0347	1.468	1.806	1.644	-0.0052
HD1	TYR	O	TYR	9	0.0081	0.0330	4.652	1.340	1.591	-0.0051
CE1	TYR	HG3	PRO	23	0.0046	0.0144	0.768	1.976	1.265	-0.0021
CE1	TYR	HB3	PRO	26	0.0043	0.0137	1.586	2.033	1.289	-0.0020
CZ	TYR	HB3	PRO	7	0.0038	0.0128	2.936	2.228	1.296	-0.0018
CE2	TYR	HB3	PRO	11	0.0050	0.0157	1.219	1.955	1.261	-0.0024
CD2	TYR	HA	PRO	36	0.0097	0.0322	0.799	1.765	1.049	-0.0055
HG2	PRO	O	PRO	34	0.0099	0.0411	0.570	1.191	1.482	-0.0066
				**Ac-Tyr-Pro-NHMe *trans***						
O	ACE	HB2	TYR	5	0.0103	0.0446	1.304	1.474	1.151	-0.0072
O	ACE	HD3	PRO	20	0.0074	0.0288	0.315	1.557	1.138	-0.0047
HA	TYR	CD	PRO	8	0.0149	0.0657	2.819	1.103	1.609	-0.0118
HA	TYR	HD3	PRO	38	0.0147	0.0590	1.026	1.073	1.210	-0.0103
HB3	TYR	HD2	PRO	9	0.0091	0.0355	0.560	1.175	1.168	-0.0056
CG	TYR	HD2	PRO	17	0.0093	0.0318	1.241	1.845	1.116	-0.0053
CD1	TYR	HD2	PRO	14	0.0088	0.0301	0.977	1.783	1.113	-0.0050
CD1	TYR	O	PRO	11	0.0044	0.0159	1.014	1.944	1.688	-0.0024

A		B		%	ρ(r_c)	∇^2ρ	ε	r_1	r_2	$H(r_c)$
CD1	TYR	N	NME	5	0.0043	0.0148	0.934	1.926	1.796	-0.0021
HD1	TYR	O	PRO	17	0.0088	0.0357	0.251	1.141	1.505	-0.0056
HD1	TYR	N	NME	5	0.0054	0.0204	0.790	1.430	1.728	-0.0029
CE1	TYR	O	PRO	7	0.0049	0.0168	0.839	1.971	1.675	-0.0025
HE1	TYR	H2	NME	6	0.0057	0.0215	0.258	1.327	1.323	-0.0031
CD2	TYR	HD2	PRO	30	0.0075	0.0250	0.710	1.787	1.140	-0.0040
HD2	TYR	O	PRO	10	0.0068	0.0264	0.337	1.258	1.560	-0.0041
O	TYR	N	NME	6	0.0085	0.0358	0.848	1.523	1.747	-0.0056
O	TYR	H	NME	15	0.0166	0.0699	0.101	1.364	0.872	-0.0126
HG2	PRO	O	PRO	34	0.0100	0.0412	11.106	1.190	1.481	-0.0066
**Ac-Tyr-Ala-NHMe *cis***										
O	ACE	HB2	TYR	9	0.0117	0.0508	0.779	1.438	1.126	-0.0082
O	ACE	O	ALA	11	0.0047	0.0175	0.801	1.651	1.720	-0.0029
O	ACE	N	NME	34	0.0052	0.0200	0.483	1.654	1.742	-0.0030
O	ACE	H	NME	15	0.0155	0.0668	0.182	1.373	0.919	-0.0120
O	ACE	H2	NME	7	0.0046	0.0189	0.452	1.644	1.302	-0.0029
O	ACE	H3	NME	6	0.0042	0.0169	0.427	1.662	1.314	-0.0026
HA	TYR	CA	ALA	7	0.0146	0.0642	2.940	1.156	1.687	-0.0115
HA	TYR	HA	ALA	60	0.0152	0.0609	0.786	1.098	1.168	-0.0107
CG	TYR	HA	ALA	35	0.0098	0.0319	0.883	1.822	1.065	-0.0055
CD1	TYR	HA	ALA	18	0.0086	0.0289	0.811	1.886	1.106	-0.0048
CE1	TYR	HB1	ALA	5	0.0051	0.0166	1.405	1.883	1.237	-0.0025
CE1	TYR	HB2	ALA	6	0.0052	0.0172	1.216	1.943	1.222	-0.0025
CE1	TYR	HB3	ALA	8	0.0051	0.0170	1.406	1.948	1.261	-0.0025
CE2	TYR	HB1	ALA	7	0.0048	0.0157	0.924	1.931	1.231	-0.0022
CE2	TYR	HB2	ALA	9	0.0050	0.0166	1.394	1.966	1.213	-0.0024
CE2	TYR	HB3	ALA	7	0.0052	0.0171	1.417	1.990	1.220	-0.0026
CD2	TYR	HA	ALA	7	0.0087	0.0289	0.811	1.902	1.112	-0.0048
HD2	TYR	O	TYR	7	0.0073	0.0291	2.714	1.404	1.582	-0.0044
**Ac-Tyr-Ala-NHMe *trans***										
O	ACE	H	NME	5	0.0147	0.0624	0.044	1.361	0.831	-0.0114
OH	TYR	H1	NME	6	0.0070	0.0295	0.385	1.595	1.192	-0.0045
OH	TYR	H2	NME	6	0.0062	0.0250	0.180	1.597	1.211	-0.0038
OH	TYR	H3	NME	5	0.0057	0.0234	0.343	1.629	1.221	-0.0036
CE2	TYR	N	NME	10	0.0053	0.0184	2.172	1.861	1.786	-0.0026
CD2	TYR	N	NME	5	0.0050	0.0177	0.972	1.898	1.743	-0.0025
CD2	TYR	H	NME	6	0.0083	0.0288	1.412	1.764	1.082	-0.0047
HD2	TYR	O	ALA	7	0.0078	0.0308	0.327	1.253	1.560	-0.0049
O	TYR	H	NME	9	0.0133	0.0563	0.117	1.427	0.973	-0.0096

Leu-Pro *cis*

A		B		%	ρ(r_c)	$\nabla^2\rho$	ε	r_1	r_2	$H(r_c)$
N	LEU	HA	PRO	9	0.0132	0.0616	0.775	1.587	1.171	-0.0100
H1	LEU	O	PRO	6	0.0278	0.1016	0.097	0.718	1.248	-0.0247
H2	LEU	O	PRO	8	0.0253	0.0880	0.176	0.794	1.310	-0.0228
H3	LEU	O	PRO	9	0.0268	0.0914	0.161	0.769	1.289	-0.0244
H3	LEU	OXT	PRO	8	0.0237	0.0862	0.162	0.803	1.317	-0.0208
HA	LEU	HD21	LEU	6	0.0116	0.0489	4.444	1.145	1.267	-0.0079
HA	LEU	HA	PRO	7	0.0142	0.0587	1.588	1.107	1.322	-0.0102
HA	LEU	C	PRO	7	0.0144	0.0601	3.347	1.037	1.562	-0.0110
HA	LEU	O	PRO	18	0.0148	0.0612	0.732	0.983	1.461	-0.0105
HA	LEU	OXT	PRO	18	0.0144	0.0594	0.720	1.010	1.445	-0.0102
HB3	LEU	HA	PRO	28	0.0095	0.0367	0.748	1.181	1.132	-0.0059
HD11	LEU	HA	PRO	7	0.0074	0.0266	0.204	1.123	1.118	-0.0043
HD12	LEU	HA	PRO	8	0.0078	0.0281	0.152	1.099	1.103	-0.0046
HD13	LEU	HA	PRO	7	0.0078	0.0281	0.131	1.101	1.106	-0.0046
HD21	LEU	HA	PRO	6	0.0054	0.0207	0.570	1.389	1.183	-0.0030
HD21	LEU	OXT	PRO	6	0.0053	0.0185	0.568	1.213	1.678	-0.0030
HD22	LEU	HA	PRO	6	0.0052	0.0199	0.496	1.362	1.201	-0.0029
HD23	LEU	HA	PRO	6	0.0052	0.0199	0.592	1.393	1.213	-0.0029
HD23	LEU	OXT	PRO	6	0.0058	0.0207	0.530	1.251	1.642	-0.0034
HG2	PRO	O	PRO	13	0.0099	0.0393	0.556	1.199	1.497	-0.0065
HG2	PRO	OXT	PRO	13	0.0099	0.0397	0.547	1.207	1.494	-0.0066
**Leu-Pro *trans***										
HA	LEU	CD	PRO	6	0.0148	0.0647	6.308	1.146	1.718	-0.0115
HA	LEU	HD3	PRO	45	0.0150	0.0605	1.076	1.128	1.132	-0.0104
HB3	LEU	CD	PRO	7	0.0091	0.0394	1.626	1.151	1.785	-0.0062
HB3	LEU	HD2	PRO	28	0.0092	0.0362	0.865	1.182	1.207	-0.0057
HG	LEU	HD2	PRO	6	0.0085	0.0321	0.365	1.149	1.171	-0.0052
HG	LEU	O	PRO	7	0.0046	0.0155	1.212	1.214	1.707	-0.0026
HD12	LEU	HD2	PRO	6	0.0067	0.0253	0.579	1.228	1.256	-0.0039
HD22	LEU	HD2	PRO	7	0.0037	0.0142	0.507	1.471	1.302	-0.0019
HD23	LEU	HD2	PRO	6	0.0049	0.0184	0.265	1.374	1.207	-0.0026
HG2	PRO	O	PRO	17	0.0098	0.0388	0.932	1.212	1.504	-0.0064
HG2	PRO	OXT	PRO	17	0.0097	0.0382	0.554	1.194	1.504	-0.0063
**Leu-Ala *cis***										
H3	LEU	OXT	ALA	6	0.0259	0.0920	0.213	0.797	1.302	-0.0237
HA	LEU	HA	ALA	34	0.0151	0.0610	0.998	1.117	1.167	-0.0106
HA	LEU	O	ALA	18	0.0146	0.0596	0.515	0.965	1.435	-0.0104
HA	LEU	OXT	ALA	24	0.0140	0.0569	0.494	0.984	1.443	-0.0099
HB3	LEU	HA	ALA	25	0.0094	0.0366	0.920	1.214	1.119	-0.0059
HG	LEU	HA	ALA	6	0.0079	0.0304	0.816	1.225	1.131	-0.0049
HD11	LEU	HA	ALA	8	0.0074	0.0269	0.340	1.157	1.120	-0.0043
HD12	LEU	HA	ALA	5	0.0085	0.0307	0.194	1.104	1.075	-0.0051

HD13	LEU	HA	ALA	7	0.0076	0.0274	0.283	1.139	1.095	-0.0044
**Leu-Ala *trans***										
-	-	-	-	-	-	-	-	-	-	

Figure S4. Images of NCI plots for the most populated cluster representatives of the peptide models. The NCI isosurfaces correspond to s = 0.5 and a color scale of −5 (blue) <sign(λ_2)ρ < 5 (red) au for HF/cc-pVTZ SMD densities with the green regions indicating weak interactions. Surface area of the isosurface elements (σ in Å^2) are also indicated.

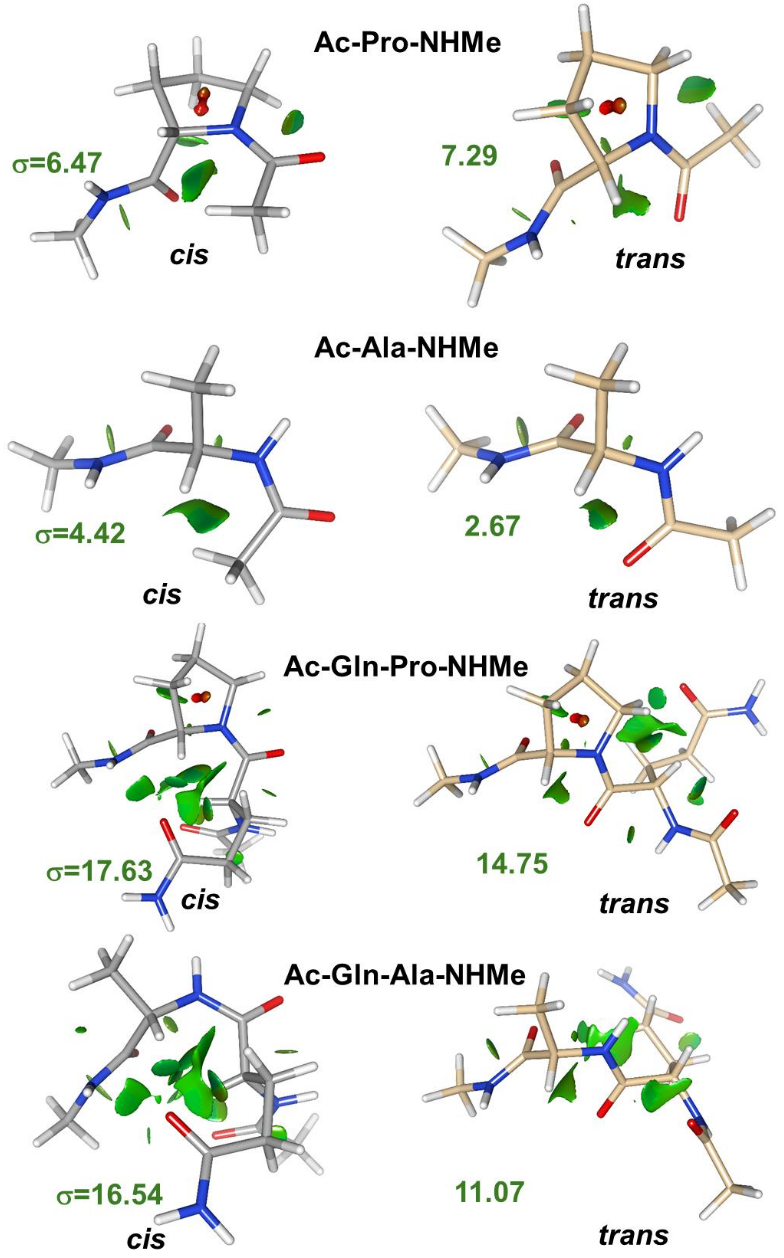

Figure S4 (cont.)

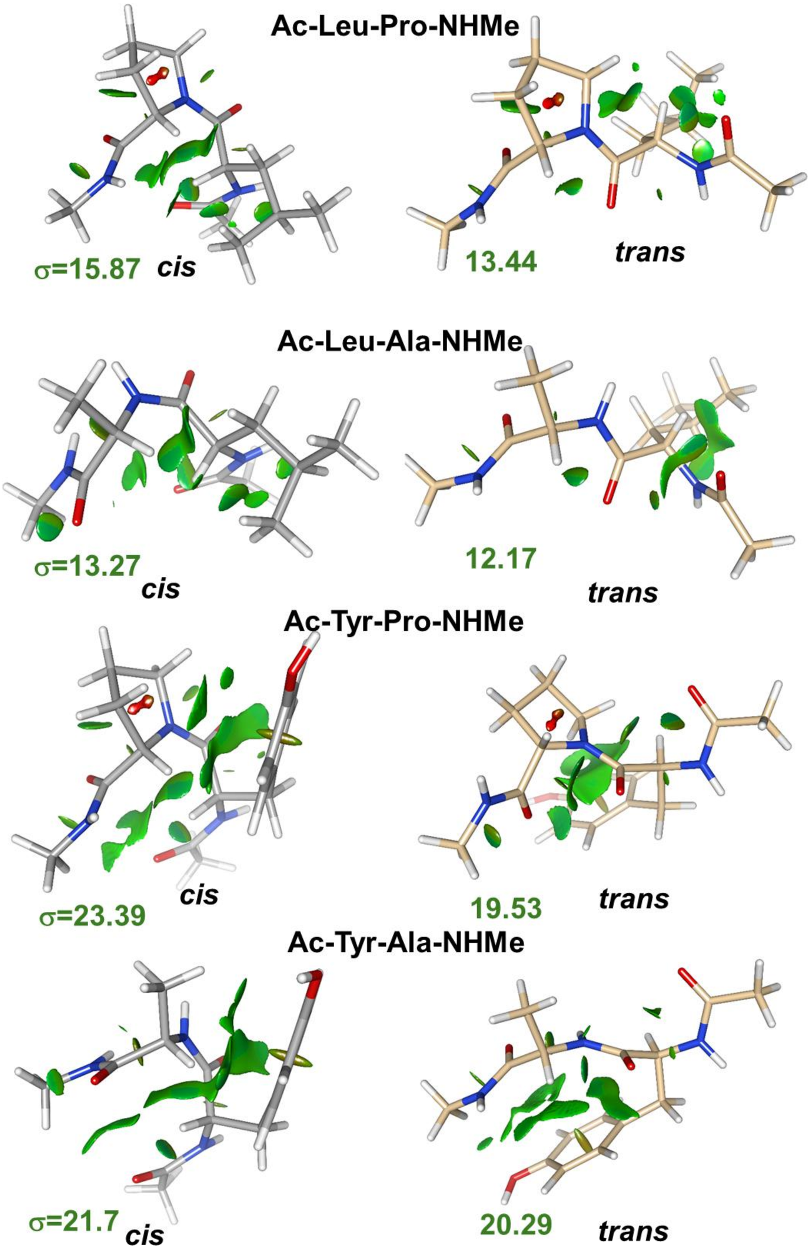

Figure S4 (cont.)

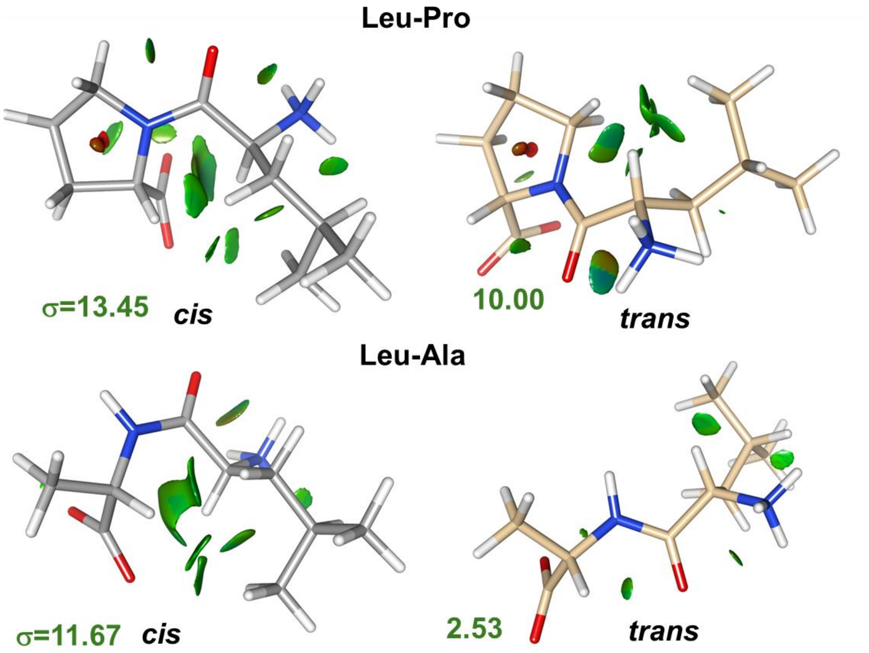

Table S6. Segregation of the average IQA terms (in kcal/mol) into short and medium range contributions to the *cis→trans* isomerization energy.

	**Atomic**	**1-2**	**1-3**	***Short***	**1-4**	**>1-4**	***Medium***
				Ac-Pro-NHMe			
$\Delta E_{T,xc}$	6.1	-0.2	-0.7	5.2	0.2	0.7	0.9
ΔE_{elec}	3.5	-10.6	-2.8	-10.0	47.3	-46.2	1.1
$\Delta E_{T,xc} + \Delta E_{elec}$	9.5	-10.8	-3.5	-4.7	47.5	-45.5	1.9
ΔE_{D3}		0.0	-0.1	-0.1	-0.1	0.8	0.7
				Ac-Ala-NHMe			
$\Delta E_{T,xc}$	1.0	-1.6	0.4	-0.2	-1.5	2.9	1.4
ΔE_{elec}	6.3	-14.4	-1.2	-9.3	44.1	-40.0	4.1
$\Delta E_{T,xc} + \Delta E_{elec}$	7.3	-16.0	-0.9	-9.5	42.6	-37.1	5.5
ΔE_{D3}		0.0	-0.1	-0.1	-0.2	1.5	1.3
				Ac-Gln-Pro-NHMe			
$\Delta E_{T,xc}$	-6.7	0.5	-0.7	-6.9	-0.8	4.9	4.0
ΔE_{elec}	8.7	-10.9	1.3	-0.9	59.8	-60.5	-0.7
$\Delta E_{T,xc} + \Delta E_{elec}$	2.0	-10.3	0.6	-7.8	58.9	-55.6	3.3
ΔE_{D3}		0.0	-0.1	-0.1	-0.1	2.3	2.1
				Ac-Gln-Ala-NHMe			
$\Delta E_{T,xc}$	-14.5	-0.3	0.3	-14.5	-1.4	8.4	7.0
ΔE_{elec}	16.4	-21.6	6.3	1.1	47.9	-44.6	3.3
$\Delta E_{T,xc} + \Delta E_{elec}$	1.8	-21.9	6.6	-13.5	46.6	-36.2	10.3
ΔE_{D3}		0.0	-0.1	-0.1	-0.2	3.4	3.1
				Ac-Leu-Pro-NHMe			
$\Delta E_{T,xc}$	-4.7	-0.5	-0.4	-5.6	-0.8	5.6	4.8
ΔE_{elec}	1.0	-3.7	2.3	-0.4	53.5	-57.2	-3.8
$\Delta E_{T,xc} + \Delta E_{elec}$	-3.7	-4.2	1.9	-6.0	52.6	-51.6	1.0
ΔE_{D3}		0.0	-0.1	-0.1	-0.2	2.7	2.6

Table S6 (cont).

	Atomic	1-2	1-3	*Short*	1-4	>1-4	*Medium*
				Ac-Leu-Ala-NHMe			
$\Delta E_{T,xc}$	-12.3	-0.3	0.7	-12.0	-1.7	6.2	4.5
ΔE_{elec}	15.1	-18.6	5.3	1.7	59.3	-56.7	2.6
$\Delta E_{T,xc} + \Delta E_{elec}$	2.8	-18.9	5.9	-10.2	57.6	-50.5	7.1
ΔE_{D3}		0.0	-0.1	-0.1	-0.2	3.3	3.1
				Ac-Tyr-Pro-NHMe			
$\Delta E_{T,xc}$	2.1	-1.0	-0.3	0.8	0.1	1.1	1.2
ΔE_{elec}	-9.5	10.6	-0.5	0.6	39.4	-44.9	-5.5
$\Delta E_{T,xc} + \Delta E_{elec}$	-7.4	9.6	-0.8	1.4	39.5	-43.9	-4.4
ΔE_{D3}		0.0	0.0	0.0	-0.1	1.6	1.6
				Ac-Tyr-Ala-NHMe			
$\Delta E_{T,xc}$	-0.7	-1.2	0.4	-1.5	-1.7	5.6	3.9
ΔE_{elec}	12.7	-27.7	4.4	-10.6	33.5	-29.3	4.2
$\Delta E_{T,xc} + \Delta E_{elec}$	12.0	-28.9	4.8	-24.1	31.8	-23.7	8.1
ΔE_{D3}		0.0	-0.1	-0.1	-0.3	2.4	2.2
				Leu-Pro			
$\Delta E_{T,xc}$	-14.6	-3.7	-0.3	-18.6	-1.2	10.4	9.2
ΔE_{elec}	6.2	1.7	-3.0	5.0	36.2	5.3	41.5
$\Delta E_{T,xc} + \Delta E_{elec}$	-5.4	-6.8	-6.2	-18.4	45.2	9.0	54.2
ΔE_{D3}		-0.2	0.5	0.3	0.5	1.3	1.8
				Leu-Ala			
$\Delta E_{T,xc}$	-13.5	-3.6	0.3	-16.8	-1.8	10.8	9.0
ΔE_{elec}	14.4	-18.0	4.9	1.3	33.0	-0.6	32.4
$\Delta E_{T,xc} + \Delta E_{elec}$	4.7	-24.6	4.8	-15.1	35.0	6.8	41.8
ΔE_{D3}		0.4	0.0	0.4	-1.2	4.0	2.8

Table S7. Summary of *cis-trans* isomerization energies (in kcal/mol) for model oligopeptides related to the dipeptides examined in this work. Experimental values correspond to Δ*G* energies derived from equilibrium constants or *cis-trans* populations. Theoretical energies correspond either to Δ*G* or to Δ*E* energies depending on the computational method.

**System**	$\Delta G_{cis\rightarrow trans}$ $\Delta E_{cis\rightarrow trans}$	**Experimental** / Theoretical **Method**	**Reference**
Ac-NHMe	-2.5 (1.5% cis)	**^{1}H NMR in water**	[1]
	-2.6	MP2/6-31G(d) calculations in water	[2]
	-2.3	B3LYP/6-311++G(3df,3pd) (gas phase)	[3]
Ac-Ala-NHMe	-2.79	B3LYP/6-311++G(d,p) & CPCM (water) HF/6-31+G(d)	[4]
	-2.1±0.1	Amber ff19SB-OPC US-PMF, T=27 ºC	This work
	-2.4 (0.2)	HF-D3/cc-pVTZ SMD (water)	This work
Ac-Pro-NHMe	-0.57 (27% cis)	**^{1}H NMR in water, T=25 ºC**	[5]
	-0.56 ($K_{trans/cis}$=2.56)	**^{1}H NMR in water, T=25 ºC**	[6]
	-0.68	B3LYP/6-311++G(d,p) & CPCM(water) HF/6-31+G(d)	[4]
	-1.0±0.3	Amber03 metadynamics	[7]
	-1.1±0.1	Amber ff99SB-TIP3P GaMD, T=27 ºC	[8]
	-1.4±0.2	Amber ff19SB-OPC US-PMF, T=27 ºC	This work
	-0.8 (0.1)	HF-D3/cc-pVTZ SMD (water)	This work
Ac-Pro-NMe_2	-0.80 (trans:cis 3.8)	**^{1}H NMR in water, T=25 ºC**	[9]
	-1.3 (trans:cis 8.8)	**^{1}H NMR in chloroform, T=25 ºC**	[9]
Ac-Gln-Ala-NHMe	-2.7±0.1	Amber ff19SB-OPC US-PMF, T=27 ºC	This work
	-2.3 (0.3)	HF-D3/cc-pVTZ SMD (water)	This work
Ac-Gln-Pro-NHMe	-1.7 (5.2% cis)	ECEPP/3 energy minimization	[10]
	-1.6±0.3	Amber ff19SB-OPC US-PMF, T=27 ºC	This work
	-1.0 (0.3)	HF-D3/cc-pVTZ SMD (water)	This work
Ac-Leu-Ala-NHMe	-2.9±0.0	Amber ff19SB-OPC US-PMF, T=27 ºC	This work
	-2.0 (0.3)	HF-D3/cc-pVTZ SMD (water)	This work
Ac-Leu-Pro-NHMe	-2.5 (1.5% cis)	ECEPP/3 energy minimization	[10]
	-1.9±0.6	Amber ff19SB-OPC US-PMF, T=27 ºC	This work
	-0.7 (0.3)	HF-D3/cc-pVTZ SMD (water)	This work

Table S7 (cont.)

**System**	**$\Delta G_{cis \rightarrow trans}$ $\Delta E_{cis \rightarrow trans}$**	**Experimental** / Theoretical **Method**	**Reference**
Ac-Tyr-Pro-NHMe	-2.7 (1.0% cis)	ECEPP/3 energy minimization	[10]
	-2.0±0.2	Amber ff19SB-OPC US-PMF, T=27 ºC	This work
	+0.1 (0.3)	HF-D3/cc-pVTZ SMD (water)	This work
Ac-Gly-Pro-NHMe	-1.01 ($K_{trans/cis}$=5.54)	**^{1}H NMR in water, T=25 ºC**	[6]
Ac-Phe-Pro-NHMe	-0.56 ($K_{trans/cis}$=2.56)	**^{1}H NMR in water, T=25 ºC**	[6]
Ac-Ala-Gln-Pro-Ala-Lys-NH_2	-1.20 (11.5% cis)	**^{1}H-NMR in water, T=23 ºC**	[11]
Ac-Ala-Leu-Pro-Ala-Lys-NH_2	-1.17 (12.0% cis)	**^{1}H-NMR in water, T=23 ºC**	[11]
Ac-Ala-Tyr-Pro-Ala-Lys-NH_2	-0.68 (24.0% cis)	**^{1}H-NMR in water, T=23 ºC**	[11]
Ala-Pro	-0.26 (39% cis)	**^{1}H NMR in water, T=25ºC, pH=7.5**	[12]
	-0.24 (40% cis)	**^{13}C NMR in water, T=25 ºC, pH=6.5**	[13]
	+0.09 (54% cis)	**^{13}C NMR in water, T=25 ºC, pH=10.5**	[13]
	-0.17	**Dynamic capillary electrophoresis in water, T=20 ºC, pH=9.5[(a)]**	[14]
	-0.9±0.1	Amber ff99SB TIP3P GaMD, T=27 ºC	[8]
Leu-Ala	-0.5±0.1	Amber ff19SB-OPC US-PMF, T=27 ºC	This work
	-0.4 (0.2)	HF-D3/cc-pVTZ SMD (water)	This work
Leu-Pro	-0.02 (49% *cis*)	**^{13}C NMR in water, T=25 ºC, pH=6.5**	[13]
	+0.34 (64% *cis*)	**^{13}C NMR in water, T=25 ºC, pH=10.5 [(a)]**	[13]
	+0.61	**Dynamic capillary electrophoresis in water, T=20 ºC, pH=9.5[(a)]**	[14]
	+0.5±0.2	Amber ff19SB-OPC US-PMF, T=27 ºC	This work
	+0.9 (0.2)	HF-D3/cc-pVTZ SMD (water)	This work
Tyr-Pro	+0.75 (78% cis)	**^{1}H NMR in water, T=25ºC, pH=7.5**	[12]
Phe-Pro	+0.72 (77% cis)	**^{1}H NMR in water, T=25ºC, pH=7.0**	[12]
	+0.68 (76% cis)	**^{13}C NMR in water, T=25 ºC, pH=6.5**	[13]
	+0.68 (76% cis)	**^{13}C NMR in water, T=25 ºC, pH=10.5**	[13]
	+1.14	**Dynamic capillary electrophoresis in water, T=20 ºC, pH=9.5[(a)]**	[14]
	+1.9±0.7	Amber ff99SB TIP3P GaMD, T=27 ºC	[8]

(a) Anionic X-Pro dominates at pH > 9

REFERENCES

1. Radzicka, A.; Pedersen, L.; Wolfenden, R., Influences of Solvent Water on Protein Folding: Free Energies of Solvation of Cis and Trans Peptides Are Nearly Identical. *Biochemistry* **1988,** *12*, 4538-4541.
2. Jorgensen, W. L.; Gao, J., Cis-Trans Energy Difference for the Peptide Bond in the Gas Phase and in Aqueous Solution. *J. Am. Chem. Soc.* **1988,** *110*, 4212-4216.
3. Thakkar, B. S.; Svendsen, J.-S. M.; Engh, R. A., Cis/Trans Isomerization in Secondary Amides: Reaction Paths, Nitrogen Inversion, and Relevance to Peptidic Systems. *J. Phys. Chem. A* **2017,** *121*, 6830-6837.
4. Kang, Y. K., Conformational Preferences of Non-Prolyl and Prolyl Residues. *J. Phys.Chem. B* **2006,** *110*, 21338-21348.
5. Beausoleil, E.; Lubell, W. D., Steric Effects on the Amide Isomer Equilibrium of Prolyl Peptides. Synthesis and Conformational Analysis of N-Acetyl-5-Tert-Butylproline N'-Methylamides. *J. Am. Chem. Soc.* **1996,** *118*, 12902-12908.
6. Taylor, C. M.; Hardre, R.; Edwards, P. J. B.; Park, J. H., Factors Affecting Conformation in Proline-Containing Peptides. *Org. Lett.* **2003,** *5*, 4413-4416.
7. Melis, C.; Bussi, G.; Lummis, S. C. R.; Molteni, C., Trans−Cis Switching Mechanisms in Proline Analogues and Their Relevance for the Gating of the 5-Ht3 Receptor. *J. Phys.Chem. B* **2009,** *113*, 12148-12153.
8. Doshi, U.; Hamelberg, D., Reoptimization of the Amber Force Field Parameters for Peptide Bond (Omega) Torsions Using Accelerated Molecular Dynamics. *J. Phys. Chem. B* **2009,** *113*, 16590-16595.
9. Siebler, C.; Maryasin, B.; Kuemin, M.; Erdmann, R. S.; Rigling, C.; Grünenfelder, C.; Ochsenfeld, C.; Wennemers, H., Importance of Dipole Moments and Ambient Polarity for the Conformation of Xaa-Pro Moieties - a Combined Experimental and Theoretical Study. *Chem. Sci.* **2015,** *6*, 6725-6730.
10. Kang, Y. K.; Jhon, J. S.; Han, S. J., Conformational Study of Ac-Xaa-Pro-Nhme Dipeptides: Proline Puckering and Trans-Cis Imide Bond. *J. Pept. Res.* **1999,** *53*, 30-40.
11. Reimer, U.; Scherer, G.; Drewello, M.; Kruber, S.; Schutkowski, M.; Fischer, G., Side-Chain Effects on Peptidyl-Prolyl Cis/Trans Isomerisation. *J. Mol. Biol.* **1998,** *279*, 449-460.
12. Grathwohl, C.; Wüthrich, K., Nmr Studies of the Rates of Proline Cis-Trans Isomerization in Oligopeptides. *Biopolymers* **1981**, 2623-2633.
13. Grathwohl, C.; Wüthrich, K., The X-Pro Peptide Bond as an Nmr Probe for Conformational Studies of Flexible Linear Peptides. *Biopolymers* **1976,** *15*, 2025-2041.
14. Schoetz, G.; Trapp, O.; Schurig, V., Determination of the Cis-Trans Isomerization Barrier of Several L-Peptidyl-L-Proline Dipeptides by Dynamic Capillary Electrophoresis and Computer Simulation. *Electrophoresis* **2001,** *22*, 2409-2415.
